# Doxorubicin impacts chromatin binding of HMGB1, Histone H1 and retinoic acid receptor

**DOI:** 10.1038/s41598-022-11994-z

**Published:** 2022-05-16

**Authors:** Rosevalentine Bosire, Lina Fadel, Gábor Mocsár, Péter Nánási, Pialy Sen, Anshu Kumar Sharma, Muhammad Umair Naseem, Attila Kovács, Jennifer Kugel, Guido Kroemer, György Vámosi, Gábor Szabó

**Affiliations:** 1 Department of Biophysics and Cell Biology, Faculty of Medicine, University of Debrecen, Debrecen, Hungary; 2 Doctoral School of Molecular Cell and Immune Biology, University of Debrecen, Debrecen, Hungary; 3 Doctoral School of Molecular Medicine, University of Debrecen, Debrecen, Hungary; 4 Department of Radiation Therapy, Faculty of Medicine, University of Debrecen, Debrecen, Hungary; 5 Department of Biochemistry, University of Colorado, Boulder, USA; 6 Centre de Recherche Des Cordeliers, Equipe Labellisée Par La Ligue Contre Le Cancer, Université de Paris, Sorbonne Université, Inserm U1138, Institut Universitaire de France, Paris, France; 7 Metabolomics and Cell Biology Platforms, Institut Gustave Roussy, Villejuif, France; 8 Pôle de Biologie, Hôpital Européen Georges Pompidou, AP-HP, Paris, France

**Keywords:** Cancer therapy, Biophysics, Cancer, Cell biology, Cell signalling, Nuclear organization

## Abstract

Doxorubicin (Dox), a widely used anticancer DNA-binding drug, affects chromatin in multiple ways, and these effects contribute to both its efficacy and its dose-limiting side effects, especially cardiotoxicity. Here, we studied the effects of Dox on the chromatin binding of the architectural proteins high mobility group B1 (HMGB1) and the linker histone H1, and the transcription factor retinoic acid receptor (RARα) by fluorescence recovery after photobleaching (FRAP) and fluorescence correlation spectroscopy (FCS) in live cells. At lower doses, Dox increased the binding of HMGB1 to DNA while decreasing the binding of the linker histone H1. At higher doses that correspond to the peak plasma concentrations achieved during chemotherapy, Dox reduced the binding of HMGB1 as well. This biphasic effect is interpreted in terms of a hierarchy of competition between the ligands involved and Dox-induced local conformational changes of nucleosome-free DNA. Combined, FRAP and FCS mobility data suggest that Dox decreases the overall binding of RARα to DNA, an effect that was only partially overcome by agonist binding. The intertwined interactions described are likely to contribute to both the effects and side effects of Dox.

## Introduction

DNA binding proteins recognize the DNA base sequence (base readout) and the structural features of DNA (shape readout) as determinants of their target sites^1^. The structural features include parameters describing intra-base pair and inter-base pair^2,3^ spatial relationships as well as minor groove dimensions (width and depth). Although they are sequence-dependent, they can also be altered by factors such as DNA supercoiling and binding of drugs. For example, the binding of intercalating drugs such as doxorubicin (Dox), increases the inter-base pair distance and untwists the DNA, which in a closed DNA molecule results also in changes in writhe. Importantly, the structural features are interdependent, e.g. changes in DNA twist affect the groove widths^4^. This implies that topological changes, while partitioning into twist and writhe, would affect the shape read-out of a particular protein.

Histone H1 and HMGB1 both bind to the linker DNA where it enters/exits the nucleosomal core particle but exert opposing effects on chromatin structure^5^. Whereas histone H1 stabilizes nucleosome structure and facilitates higher order chromatin folding^6^, binding of HMGB1 enhances nucleosome sliding and decompacts chromatin^7^. Consequently, histone H1 represses transcription, contrasting with HMGB1, which promotes the interaction of transcription factors (TFs) with DNA. Although both proteins bind to DNA without sequence specificity, their binding is influenced by DNA structure. Histone H1 preferentially binds to superhelical plasmid DNA in vitro, a fact that may be explained by its increased potential to form multiple H1-DNA contacts on plectonemically wound DNA^8^. HMGB1 preferentially binds to pre-bent DNA, supercoiled DNA, damaged DNA, hemicatenanes and catenanes, four-way junctions and other non-B DNA structures in vitro^9^*.* Upon binding, HMGB1 further bends the DNA towards the major groove by an angle of about 77°. This bending results from the intercalation of three hydrophobic amino acids through the minor groove^10,11^.

In sharp contrast to the aforementioned structural proteins, TFs recognize and bind specific sequences positioned immediately upstream of the transcription start site or far away from the promoter, to modulate gene transcription^12^. With the exception of pioneer factors, TFs are able to engage with their binding sites only within nucleosome-free regions. Upon TF-DNA interaction, DNA undergoes deformations such as bending and/or kinking to accommodate the TF^13^. Since bending demands energy, TFs preferentially bind to pre-bent DNA structures or DNA that is easily deformable^14^.

Retinoic acid receptor (RAR) is a member of the nuclear receptor (NR) family of TFs, the activation of which is ligand-dependent. RAR requires heterodimerization with retinoid X receptor (RXR) for proper transactivation of its target genes. In chromatin, RAR-RXR heterodimeric complexes recognize a specific sequence called retinoic acid response element (RARE) composed of a direct repeat (DR) of AGGTCA sequences separated by a spacer of either 1, 2 or 5 bases, termed DR1, DR2 and DR5, respectively. Binding occurs through the major DNA groove^15^.

The mechanism of action of RAR and other nuclear receptors can be explained by the molecular switch model. In the presence of its agonist (all-trans RA, ATRA, or 9-cis RA), RAR recruits coactivator complexes and activates transcription, whereas in the absence of an agonist it binds corepressors with histone deacetylase activity^16^. Previously, we have shown by fluorescence correlation spectroscopy (FCS) measurements that in contrast with the original static model, RAR (as well as RXR) binding is dynamic. Its diffusion in the nucleus can be described by a model assuming a fast, freely diffusing component corresponding to monomers or small complexes, and a slow, chromatin-bound component. Agonist treatment increased the slow fraction in a coactivator-dependent manner and enhanced dimerization with RXR^17–20^. In view of our ChIP-seq data, ligand treatment can enhance the binding of NRs to their response elements on DNA^18,21^. The genes controlled by the RAR-RXR heterodimer include topoisomerase IIβ (TopIIβ), which is suppressed by the binding of the heterodimer to a RARE positioned 907 bp downstream of the TopIIβ transcription start site^21^ linking RAR signalling and Dox-induced TopIIβ cleavage complexes to cardiotoxicity, a major hazard when applying the drug for the treatment of human malignances^22^. Whether and how Dox may also influence the DNA-binding properties of RAR has not been determined before.

These studies have been motivated by our previous observation that the toroid nucleosomal superhelicity of genomic DNA is a powerful impediment to the intercalative binding of small molecules to native chromatin, from which we have concluded that the structural constraint imposed by the nucleosome on the DNA wrapped around it, rather than diffusional access limitations, are likely to control ligand binding^23^. In contrast, the nucleosome-free regions including the internucleosomal linker DNA were readily bound by the same dyes, meaning that the plectonemic superhelicity of these regions easily adapts to accommodate such ligands^23^. These findings spurred us to investigate how small molecule intercalators influence the binding of proteins known to target these regions, including structural constituents of chromatin, high-mobility group protein B1 (HMGB1) and the linker histone H1, as well as a sequence-specific transcription factor, RARα.

DNA binding of these proteins was studied in live cells, using FRAP that gives information about the average diffusion properties within a larger area, over distances of a few µm-s, and FCS, which reflects local diffusion behavior and binding within a diffraction-limited detection volume smaller than 1 µm^3^ within a lateral radius of ~ 200 nm. Binding of all of these proteins was insensitive to topological relaxation itself. On the other hand, intercalators, including Dox, an anthracyclin widely used in cancer chemotherapy, had a robust and biphasic effect by interfering with H1 binding and thereby facilitating the association of HMGB1 with DNA at low-to-moderate Dox concentrations, and by directly competing with all the proteins investigated at higher drug concentrations. Our findings provide insights into the interplay of ligand binding to native chromatin and add further items to the long list of the cellular effects of anthracyclins. Particularly intriguing among these is the overall reduction of RAR binding since the retinoic acid signaling pathway has emerged recently as a major player in doxorubicin-induced cardiotoxicity (DIC)^24,25^.

## Results

### The anticancer drug Doxorubicin impacts HMGB1 distribution and mobility in the nuclei of live cells

**Figure 1:**
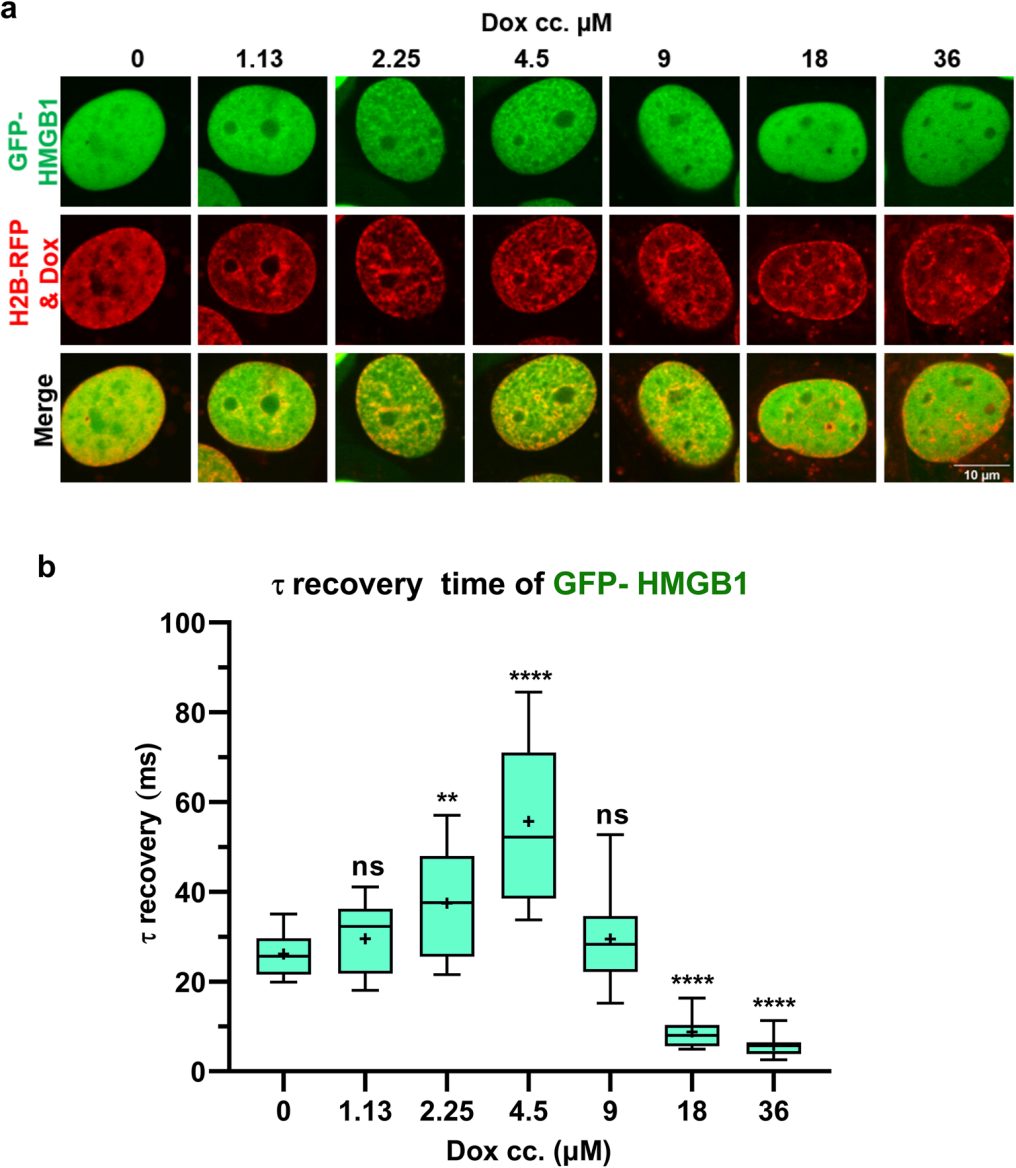
*Doxorubicin affects HMGB1 dynamics in a concentration dependent manner*. (**a**) Representative nuclei of U2OS^2FP^ cells treated with the indicated concentrations of Dox for 2 h. (**b**) GFP-HMGB1 recovery times of Dox-treated cells as measured by point FRAP. The graph shows one representative experiment of three repeats. In this as well as in the other figures, box-and-whiskers plots represent 10th, 25th, 50th, 75th, and 90th percentiles; + , mean value. One-way ANOVA with post hoc Dunnett’s test was used to calculate significance compared to 0 Dox (**p < 0.01, ****p < 0.0001). Data were analyzed using GraphPad Prism 8.01.

Treatment of U2OS^2FP^ cells stably expressing GFP-tagged HMGB1 and RFP-tagged H2B with Dox caused a loss of GFP-HMGB1 from nucleoli while its distribution at the chromatin became more structured (Fig. 1a). However, at a Dox concentration of > 9 µM, the structured distribution in the chromatin was lost and the localization of GFP-HMGB1 became more diffuse. This biphasic effect on chromatin distribution was also reflected by the mobility of GFP-HMGB1 measured by point FRAP. The recovery time increased with increasing Dox concentration peaking at about 50 ms for samples treated with 4.5 µM and then declined at higher Dox concentrations (Fig. 1b and Fig. S1). Similar observations were made following treatment with another intercalating drug, ethidium bromide (EBr) (Fig. S2).

Drug intercalation to DNA increases the base pair rise while reducing the helix twist by an angle dependent on the intercalator molecule. This decrease in helix twist translates into an overall reduction in DNA twist, which is compensated by an increase in writhe within the chromatin loops^23^. To elucidate the possible contribution of superhelicity to the recovery profiles generated above (Fig. 1b and Fig. S1), we induced topological relaxation by nicking agents.

### DNA nicking had no effect on HMGB1 binding in live cells

**Figure 2:**
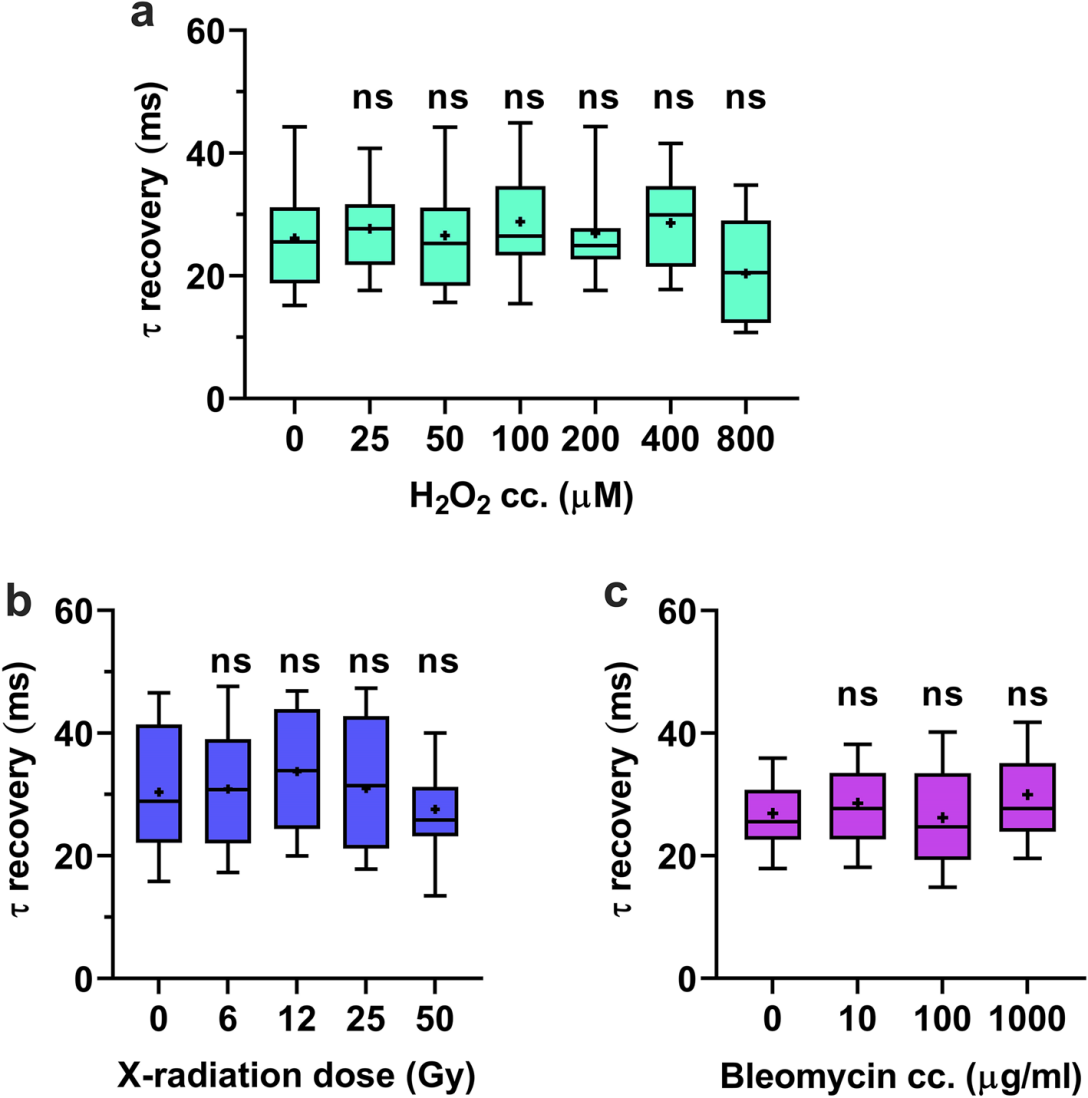
*Relaxation of supercoiling by nicking does not affect chromatin binding of HMGB1*. U2OS^2FP^ cells were treated with (**a**) 25, 50, 100, 200, 400 and 800 µM H_2_O_2_ for 20 min, or (**b**) with 10, 100 and 1,000 µg/ml bleomycin for 2 h, or (**c**) irradiated with 6, 12, 25 and 50 Gy X-ray, then the mobility of GFP-HMGB1 was measured by point FRAP. Graphs show one representative experiment of three repeats. The statistical test used is one-way ANOVA (α < 0.05).

In view of the well-known winding effect of intercalators^26,27^ (and refs therein), we tested the possible role of internucleosomal superhelicity in determining HMGB1 binding in vivo. DNA supercoiling was relaxed by treating live U2OS^2FP^ cells with hydrogen peroxide (H_2_O_2_), bleomycin or X-ray irradiation, and GFP-HMGB1 binding to DNA was evaluated by point FRAP. Given the short time interval between nicking and measurement, we expected that the breaks would still be unrepaired, or even if they were, the original levels of internucleosomal superhelicity would not have been re-established. Thus, if HMGB1 binding were sensitive to supercoiling, relaxation would affect its binding. Strikingly, none of these agents caused a change in FRAP recovery rate (Fig. 2), suggesting that intercalators influence HMGB1 binding directly rather than indirectly by changing twist-writhe partitioning.

The lack of effect of DNA supercoiling on HMGB1 binding in live cells was surprising since there was a slight but clear difference between the topological forms of plasmid DNA in their ability to bind recombinant HMGB1 (rHMGB1), when all the forms were simultaneously present (Fig. S3). As seen on the gel image, the migration of supercoiled plasmid DNA was retarded by as low as 60:1 rHMGB1-to-plasmid molar ratio and this retardation became more pronounced with increasing amounts of rHMGB1. Migration of linear DNA was not affected by up to a somewhat higher (100:1) protein-to-plasmid ratio, indicating a slightly lower rHMGB1-binding affinity for linear as compared to supercoiled DNA.

### Dox decreases the binding of HMGB1 to supercoiled plasmid DNA

**Figure 3:**
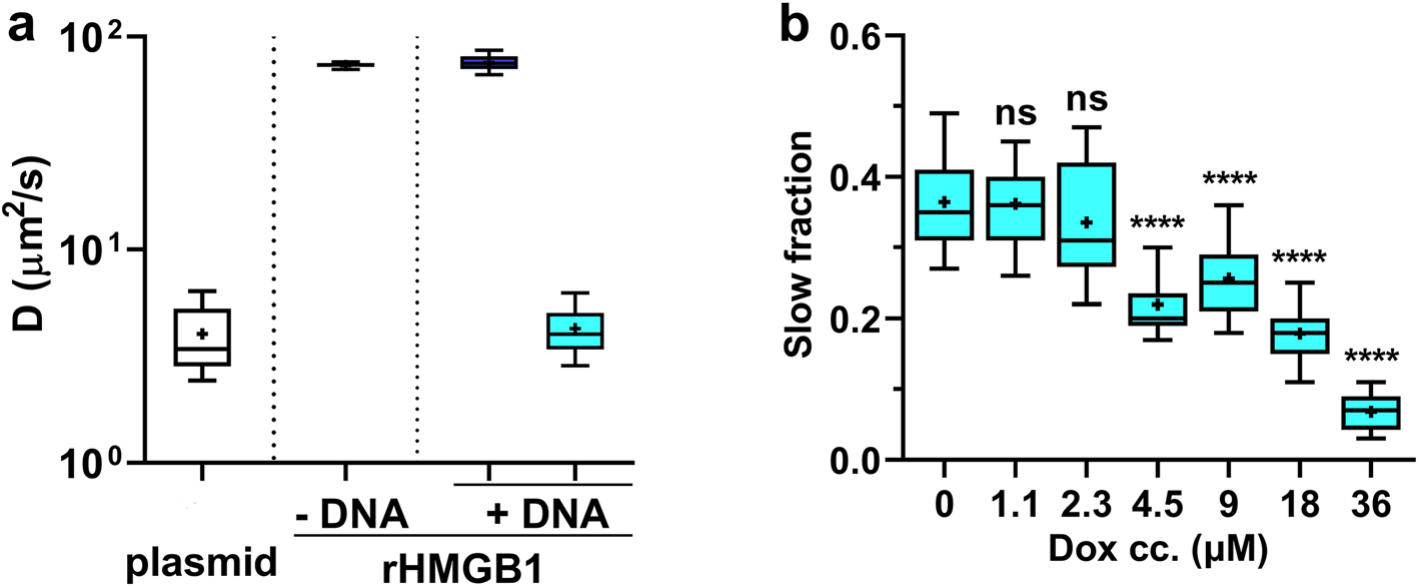
*Dox decreases the binding of rHMGB1 to plasmid DNA*. (**a**) Diffusion coefficient of SYBR Gold stained superhelical plasmid DNA (4700 bp) and of Alexa-647 labelled rHMGB1. The protein had a single diffusing species when alone in the solution and two in the presence of the plasmid DNA as measured by FCS. (**b**) Fraction of rHMGB1 bound to supercoiled plasmid DNA in the presence of varying concentrations of Dox. One-way ANOVA with post hoc Dunnett’s test was used to calculate significance ****p < 0.0001.

The diffusion constant (D) of rHMGB1 in solution was 73 µm^2^/s as determined by FCS. In the presence of native plasmid DNA, two diffusing components were observed: a fast component corresponding to freely diffusing rHMGB1 with D ≈ 76 µm^2^/s and a slow component with D ≈ 4 µm^2^/s, which was interpreted to be DNA-bound HMGB1 based on the fact that the diffusion constant of Sybr Gold stained native plasmid DNA was ≈ 3 µm^2^/s (Fig. 3a). Addition of Dox at increasing concentrations led to a monotonous decrease in the fraction of HMGB1 bound to plasmid DNA (Fig. 3b). This finding suggests that Dox may directly compete with HMGB1 binding.

### Dox displaces histone H1 from chromatin in live cells

**Figure 4:**
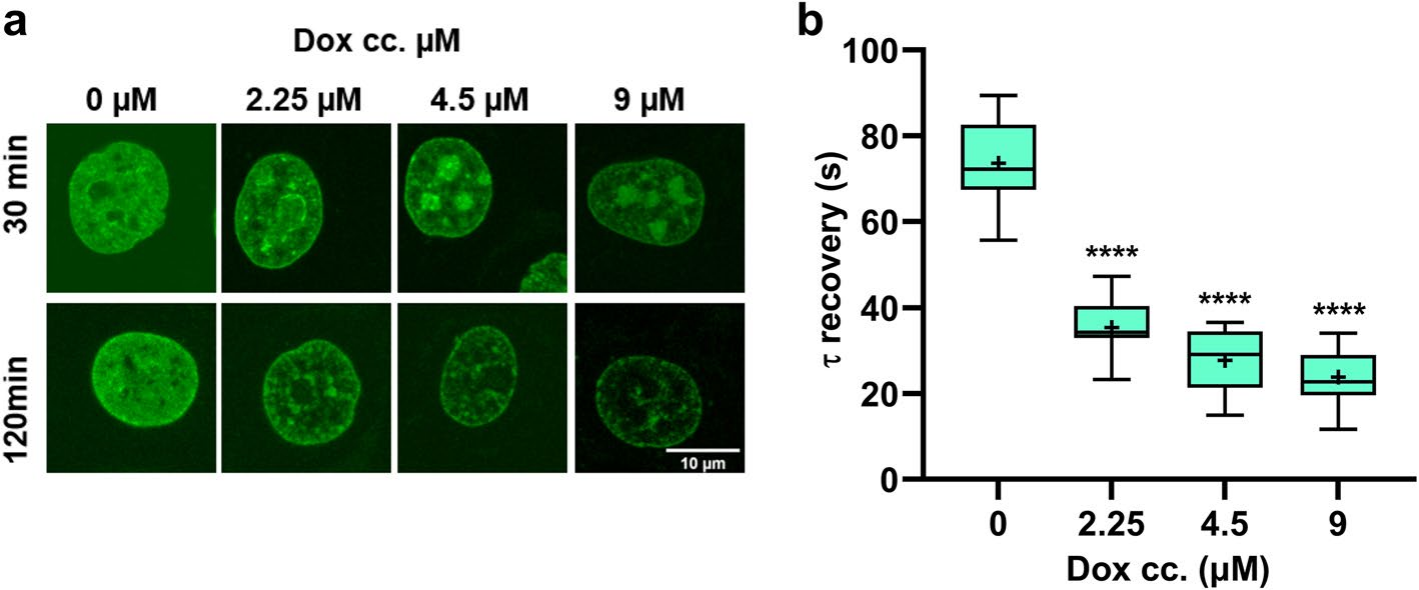
*Doxorubicin affects histone H1c binding to genomic DNA in a dose- and time-dependent manner*. (**a**) Representative images of H1c-GFP expressor HeLa cells treated with Dox for 30 (top row) or 120 min (bottom row). (**b**) FRAP analysis of H1c-GFP intranuclear mobility without Dox treatment and after 30 min treatment with different concentrations of the drug. One-way ANOVA with post hoc Dunnett’s test was used to calculate significance (****p < 0.0001).

Previous studies have shown that HMGB1 competes with histone H1 for binding to the linker DNA near the nucleosome dyad^5,28^. Furthermore, Daunomycin, a drug structurally similar to Dox can evict the linker histone variant H1.1 from chromatin^29^. H1.1 is one of the human somatic histone H1 variants the expression of which is replication-dependent. However, unlike H1.2, H1.3, H1.4 and H1.5 that are ubiquitously expressed, expression of H1.1 is limited to certain tissues^30^. To determine if Dox had a similar effect, HeLa cells expressing GFP tagged H1c, a mouse ortholog of human H1.2, were treated with increasing concentrations of Dox and the distribution and mobility of the histone were assessed up to the Dox concentration still providing sufficient H1c-GFP fluorescence intensity for the FRAP measurements. The effect of Dox on H1c binding *in viv*o was both time and concentration dependent. Within 30 min, large-scale Dox-induced eviction of H1c-GFP from chromatin and its redistribution towards nucleoli was observed (Fig. 4a). This effect was accompanied by an increased mobility as measured by strip FRAP (Fig. 4b). After 2 h of Dox treatment, the nucleolar component disappeared and there was a generalized loss of H1c-GFP from the cell (Fig. 4a). Similarly, EBr caused displacement of H1c-GFP from chromatin (Fig. S4). The displacement of histone H1 from DNA likely increases the number of available HMGB1 binding sites in the genomic DNA, thus explaining its recruitment to chromatin and the slower recovery rates in the first phase of the bimodal response (Fig. 1b and Fig. S2).

### Dox affects the binding of RARα to DNA

**Figure 5:**
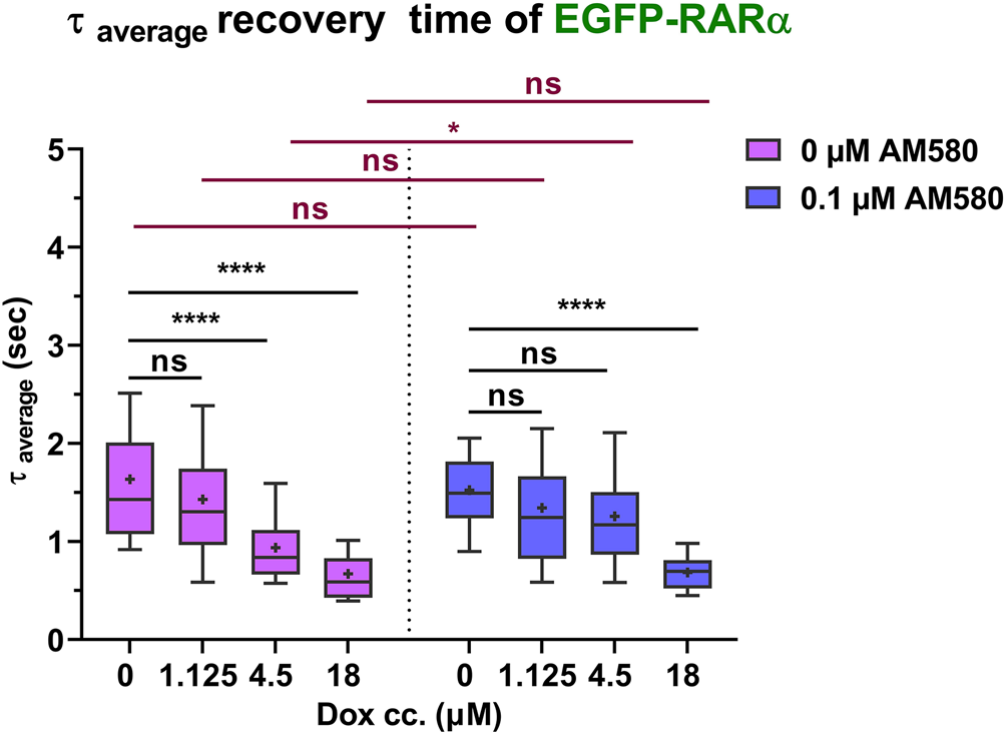
*Dox reduces RARα binding to DNA *in vivo. Average recovery times of EGFP-RARα derived from strip FRAP experiments in cells treated with different concentrations of doxorubicin (0, 1.125, 4.5, 18 µM) in the presence or absence of the RAR-specific agonist AM580 used at 0.1 µM. Two-way ANOVA with Tukey’s multiple comparison test was used to calculate significance; *p < 0.05; **p < 0.01; ***p < 0.001; ****p < 0.0001.

To assess if intercalators affect the DNA binding of TFs, we extended our studies to the nuclear receptor RARα. RARα binds to DNA in a sequence-specific manner via its zinc-finger motif not involving intercalating amino acids. Strip-FRAP was used to analyse the average mobility of RARα over distances of several micrometers. Our FRAP data could be fitted to a biexponential model function (see Eqs. 2–5; for FRAP curves see Fig. S5). Figure 5 shows the diffusion behaviour in terms of the weighted average of the recovery times, *τ*_*average*_; the detailed behaviour of the individual components is shown in Fig. S6. With increasing doses of Dox, the overall mobility of RARα increased; i.e., its DNA-binding decreased as suggested by the reduced *τ*_*average*_. At 1.125 µM Dox concentration it was only slightly reduced, whereas at 4.5 µM it decreased from 1.43 to 0.94 s. A further reduction in RARα binding was observed at 18 µM concentration, to 0.67 s. The mobile pool (Fig. S6d) decreased slightly, from 98% for the control to ~ 93% at 18 µM Dox. Treatment with the specific RARα agonist AM580 (used at 0.1 µM concentration) ameliorated the reduction in RAR binding up to 4.5 µM Dox concentration, while *τ*_*average*_ was reduced to 0.69 s upon treatment with 18 µM (Fig. 5, right). For further details see Fig. S6.

To assess if the observed increase in RAR mobility could be due to a change of microviscosity in the nucleus, we applied FRAP to the EGFP dimer, an inert molecule not binding to DNA. Its recovery time was not affected by Dox treatment (see Fig. S7) indicating that microviscosity was not significantly altered. Thus, the FRAP-derived increase of mobility of EGFP-RARα is indeed due to its decreased chromatin binding.

**Figure 6:**
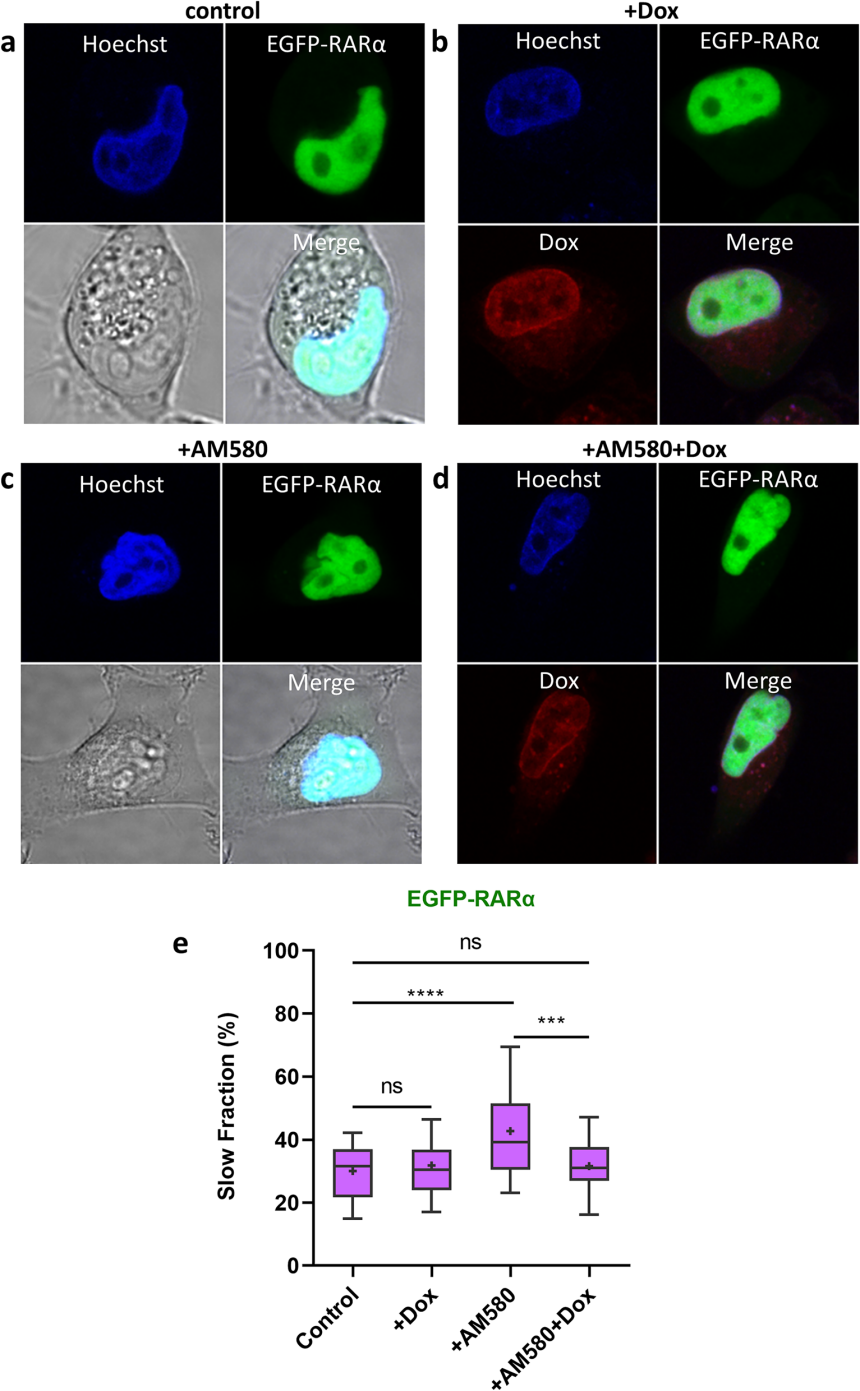
*Doxorubicin reduces agonist-induced DNA-binding of RARα*. (**a**–**d**) Representative confocal images of the distribution of stably expressed EGFP-RARα in HeLa cells are shown along with Dox staining (4.5 µM) and Hoechst 33,342 (10 µM) counterstaining patterns. RAR-specific agonist AM580 was applied at 0.1 µM where indicated. (**e**) Fraction of the slow component of EGFP-RARα determined from FCS measurements of HeLa cells; values for the untreated control, for cells treated with 4.5 µM doxorubicin, 0.1 µM RAR-specific agonist AM580, or both, as indicated in the figure. Two-way ANOVA with Tukey’s multiple comparison test was used to calculate significance; ***p < 0.001; ****p < 0.0001; ns, not significant.

To reveal the subcellular distribution of the chromatin and EGFP-RARα, confocal images were taken. The distribution of EGFP-RARα exhibited only mild spatial variations, whereas that of Dox was more heterogeneous displaying very bright and dark areas, irrespective of treatment with an agonist ligand (0.1 µM AM580) (Fig. 6a–d).

The uneven distribution of Dox prompted us to probe the local mobility of EGFP-RARα with FCS, an approach capable of distinguishing ligand-specific RARα binding through the fraction of the slow component of the autocorrelation curves^18^. The slow, DNA-bound fraction of RARα in control and in ligand and/or Dox-treated cells is shown in Fig. 6e. Treatment with 4.5 µM of Dox alone had no effect on the proportion of the slow fraction, whereas the RAR-specific agonist AM580 (used at 0.1 µM) enhanced it from 30 to 44%, similar to our earlier observations^18^. The ligand induced increase in the slow fraction was reduced if the cells were also treated with Dox. (For further details see Fig. S8). We also measured the local mobility of the EGFP dimer by FCS. The FCS-derived diffusion coefficient displayed no significant change upon 4.5 µM Dox treatment either (Fig. S8c).

### DNA nicking has no effect on RARα diffusion

We have also evaluated if DNA superhelicity has an effect on the binding of RARα in vivo, using 200 µM H_2_O_2_ as a nicking agent to relax the supercoiled genomic DNA. As a negative control, the diffusion of the EGFP dimer, transiently expressed in HeLa cells, was evaluated. The diffusion coefficient of the EGFP dimer (D ≈ 20 µm^2^/s, see Supplementary Figs. S8a and S9) was unaffected by H_2_O_2_ treatment. This implies that H_2_O_2_ did not alter the structure of the chromatin in a manner that would influence the diffusion rate of a nuclear protein having no binding site in chromatin. Next, we analysed the effect of H_2_O_2_ on RARα binding. Treatment with H_2_O_2_ did not change either the diffusion coefficient (Fig. S9b, c) or the proportions of the fast and slow fractions of RARα (Fig. S9d). H_2_O_2_ had no significant effect on these parameters in AM580 treated cells either (Fig. S9b–d). Thus, we conclude that DNA relaxation by nicking does not affect the RAR-binding capacity of the chromatin^31^.

## Discussion

Here we report that Dox intercalation into DNA in vivo affects HMGB1 binding to DNA in a bimodal fashion, i.e. increasing binding of the protein at lower concentrations, and causing a drastic reduction at concentrations higher than 9 µM (Fig. 1). The phase of increased HMGB1 binding is well explained by histone H1 displacement from DNA in view of the facts that both H1 and HMGB1 compete for the linker DNA near the nucleosome dyad^5^. In line with this notion, histone H1 exhibits higher sensitivity to intercalator binding than HMGB1, i.e. H1 is readily displaced from chromatin by both Dox and EBr at drug concentrations where HMGB1 is still bound to DNA (compare Fig. 4 with Figs. 1 and Fig. S2).

The reduction in HMGB1 binding to DNA at high Dox concentrations (Fig. 1b) could be due to intercalator induced DNA distortion, or alternatively, to competition between Dox and HMGB1 for binding sites on DNA. Dox carries two DNA binding moieties, the anthraquinone moiety which intercalates between adjacent G-C base pair steps, and the amino sugar which is positioned in the DNA minor groove^32^. Intercalation of the anthraquinone moiety increases the base pair rise to 5.2 Å and reduces the helical twist at the site of intercalation. In vivo*,* unwinding of the DNA due to intercalation would be compensated for by positive writhing within the closed chromatin loops without change in the linking number, in line with the equation *ΔLk* = *ΔTw* + *ΔWr*. The level of the compensatory positive torsion would increase with the number of intercalated molecules. This intercalator-induced change in torsion together with that induced by ongoing replication and transcription would trigger topoisomerase activity to resolve it, as reviewed in^33,34^. However, at high concentrations (> 9 µM), Dox would inhibit the binding of the topoisomerase to DNA (as reviewed in^35^), thus leading to accumulation of positive writhe. Although some of the positive torsion may be annihilated by the negative torsion stored in nucleosome-bound DNA following destabilization of nucleosomes^36,37^, this may reduce but not completely abrogate positive torsion. The positive writhe would thus hinder the binding of HMGB1, which involves intercalation^38^. This argument would be in line with the monotonous reduction in HMGB1 binding to covalently closed plasmid DNA in the presence of Dox (Fig. 3b). However, the lack of any detectable change in HMGB1 binding upon nicking treatments suggests that the contribution of the above mechanism to the decreased binding may not be significant. Thus, local distortion of DNA conformation or competition of the two ligands for DNA are suggested to account for the results of Fig. 3b.

Regarding the latter type of interaction, the binding of Dox and HMGB1 to DNA overlap in two aspects: both involve intercalation into DNA as well as minor groove binding. HMGB1 possesses two DNA binding domains, box A and B, which are connected by a short linker to an intrinsically disordered, acidic C-terminal tail^39^. The A and B domains form an “L”-shape with a concave DNA binding surface. The binding of HMGB1 to DNA occurs through the DNA minor groove and induces a bend towards the major groove. Additionally, hydrophobic amino acids phenylalanine (Phe38) on box A, phenylalanine (Phe1103) and isoleucine (Ile122) on box B partially intercalate between base pairs following DNA binding, and unwind the DNA^38^. This overlap in the Dox and HMGB1 binding to DNA is bound to create grounds for competition.

Even at the highest concentration of EBr used (100 µM), HMGB1 binding to DNA exceeded that of the control, as seen in the microscopy images and also through the point FRAP profiles (Fig. S2). This is in sharp contrast to Dox, which at drug concentrations > 9 µM reduces HMGB1 binding to DNA. This effect may be attributed to the lower uptake of EBr by live cells due to its positive charge, which means that the amount of dye intercalated in DNA is much lower than in the case of Dox. Doxorubicin is known to accumulate in cells attaining a higher intracellular concentration as compared to the extracellular milieu. Another factor in the higher efficiency of Dox to suppress HMGB1 binding may be the presence of the amino sugar positioned in the DNA minor groove.

Relaxing DNA writhe in chromatin loops by nicking via X-ray irradiation, treatment with H_2_O_2_ or bleomycin did not yield any observable effect on the binding of HMGB1 in vivo (Fig. 3). This is in contrast with the well documented preference of HMGB1 binding to supercoiled over relaxed DNA in vitro (Fig. S3; see also^38^). At the dosage used in these experiments, X-ray irradiation is estimated to cause 6000–50,000, while H_2_O_2_ provokes 15,000–36,000 single strand breaks per nucleus^40,41^ (see also Fig. S11). Even if a fraction of single strand breaks may be repaired within minutes^42^, many of the breaks would remain unrepaired given the short time interval between nicking and measurement. Moreover, it appears unlikely that the initial level of supercoiling could be restored by RNA and DNA polymerization according to the twin supercoil model^43^ in these circumstances. The fact that HMGB1 binding in vivo was not affected by DNA nicking implies that the protein binds similarly to relaxed and supercoiled DNA when it is the only conformation available. Furthermore, the overall negative supercoiling of internucleosomal DNA may be much smaller than that of the supercoiled plasmid. Therefore, the topological preference of the protein emphasized earlier based on in vitro data^38^ is probably irrelevant in the cellular context.

Other groups have observed nuclear to cytoplasmic translocation or extracellular secretion of HMGB1 from immune cells following ionizing radiation or H_2_O_2_ treatment, at doses lower than those used here^44,45^. This, however, occurred 3–24 h after exposure, i.e. after most of the single strand breaks would have been repaired and thus cannot be directly related to changes in superhelicity or H1 binding.

The evidence presented here suggests that supercoiling may not affect HMGB1 binding in vivo, or any effect of supercoiling may be overshadowed by interactions involving the chromatin environment, as follows. In the context of the general rules governing ligand binding to chromatin, although the nucleosome-constrained topology and the interrelated structural features of the DNA pose a powerful obstacle of ligand access to DNA^23^, binding to the flexible internucleosomal DNA regions is, in general, not determined by DNA topology; here competition among the ligands or distortion of the DNA structure around the bound anthracyclin seem the dominant controlling factors. The concentration dependent influence of Dox on HMGB1 binding has not been recognized before and may contribute to the effects and side-effects of this medically relevant anthracycline.

We also analyzed how Dox treatment affects the DNA binding of a sequence-specific TF, RARα. This TF is unlikely to have an effect on the balance of H1 and HMGB1 genome-wide, i.e. its effects must be restricted to the regulatory regions of RAR responsive genes. On the other hand, upon ligand dependent RAR binding, HMGB1 may be recruited at these sites while H1 is displaced^46^. This exchange would be facilitated by Dox at low drug concentrations where it evicts H1 without affecting HMGB1 binding, leading perhaps to augmented RAR binding. However, such an effect, if it occurred, was apparently overcome by the decrease in DNA binding, seen also in the absence of the ligand (Fig. 5).

We measured the mobility of EGFP-RARα on a distance scale of a few micrometers, averaged for larger areas, with FRAP (Fig. 5, Fig. S6), and on a shorter distance scale of a few hundred nanometers, locally, with FCS (Fig. 6e, Fig. S8). The FRAP recovery curves likely reflect the extent of overall DNA binding, while being also influenced by the diffusional constraints. The latter may be altered upon Dox treatment that is known to elicit gross morphological changes in the nucleus due to histone eviction and consequential aggregation^36^. With both methods we could detect a fast and a slow component, and with FRAP an additional fraction appearing as “immobile” over the distance scale of FRAP. The FRAP-detected fast and slow components refer to molecules that leave the few-micrometer wide ROI during the time course of the measurement; thus, both are diffusible and even the slow component can only be transiently bound to DNA. In view of our earlier FCS studies in this system^17–20,47^, the fast FCS component may likely be identified with the fast component of FRAP, which probably represents the freely diffusing RARα species or what is bound to DNA for very short dwell times, ≈ 1 ms according to our FCS fits (Fig. S10). The slow FCS component likely corresponds to transiently DNA-bound RARα (dwell time in the order of several hundred milliseconds) as well as stably bound RARα moving slowly together with the chromatin. The slow FCS component is likely a mixture of the slow and “immobile” populations detected by FRAP. Similar slow diffusion coefficients were detected by FCS for other DNA-binding proteins as well^47–49^.

Our most noteworthy observation via FRAP was that Dox increased the average mobility of RARα (Fig. 5). This increase was confirmed to be due to shorter dwell times spent DNA-bound rather than to a change of microviscosity, since the diffusion coefficient of the EGFP dimer was not affected (Fig. S7). On the other hand, Dox slightly enhanced the immobile fraction (decreased the mobile fraction) in a dose-dependent manner (Fig. S6d). This is in line with the decrease of the FCS-determined slow diffusion coefficient attributed to DNA-bound receptors (Fig. S8b). Thus, Dox seems to have a dual effect on the DNA-binding of RARα. First, there is a prominent Dox-induced decrease of overall RARα binding to the DNA, and second, a much smaller fraction of RARα displays decreased mobility. This latter minor component may be due to a stronger DNA-binding of the TF to the dehistonized DNA visible in the Dox-reorganized nuclei^36^.

Previously, we have shown by FCS, FCCS and FRET that the RAR agonist AM580 augmented heterodimerization of RARα with RXRα, increased the proportion of the slow fraction and enhanced its DNA-binding^18–20^. Here, treatment with saturating concentrations (0.1 μM) of AM580 partly counteracted the effect of Dox on the average mobility of RARα; i.e., the reduction of the average FRAP recovery time was mitigated in the presence of the ligand at a Dox concentration of up to 4.5 µM, which is within the range of the therapeutic doses^50^. Our FCS measurements reveal that Dox, used at a concentration easily reached in the tissues during chemotherapy^37^, overcomes the ligand-induced increment of the slow fraction, while not affecting it in the absence of the ligand (Fig. 6e). Thus, Dox decreases ligand-independent as well as ligand-dependent RARα binding, based on FRAP and FCS data, respectively.

The reduction in binding of RARα following Dox treatment may suggest yet another mechanism by which Dox may exert its cytotoxicity. As the binding of RARα to DNA does not involve intercalation, and there was no effect of nicking on RARα binding (Fig. S9), the reduction may be explained by either direct competition between Dox and RAR for the binding sites on the DNA or the drug’s effect on its structural features. For example, Dox intercalation increases the stiffness of the DNA^51^ and hence may decrease DNA deformability that is required for optimal TF binding^15^.

Despite the high efficacy of Dox in treating malignancies, its therapeutical use is limited by cardiotoxicity^52^. Several studies have linked Dox induced cardiotoxicity (DIC) to topoisomerase poisoning, while others emphasize the role of ROS production^24^. RARγ was recently shown to bind to the Top2β gene at a retinoic acid response element (RARE) positioned downstream of the transcription start site, thereby repressing gene transcription in the presence of ATRA. However, in cells expressing the nonsynonymous SNP variant RARγ-S427L, this transrepression is compromised, thus aggravating DIC^21,25^. Indeed, correction of this RARγ-S427L SNP by genetic engineering reduced the cytotoxicity of Dox^53^. Based on these findings, we propose that Dox inhibition of RAR binding to chromatin may contribute to cardiotoxicity. We speculate that a separate, rather than combined, administration of ATRA and Dox could mitigate DIC, while not affecting Dox cytotoxicity in cancer cells where the role of RAR signalling in Top2β control may not be relevant or tight.

In summary, Dox, at ≤ 4.5 µM concentration, augments HMGB1 binding by causing H1 eviction, while strongly inhibiting HMGB1 binding at higher doses also reached in blood plasma levels during chemotherapy. In addition, Dox reduces RARα binding, thus affecting a TF that contributes to the pathogenesis of DIC.

## Materials and methods

### Cell culture

HeLa cells stably expressing GFP-tagged histone H1 (HeLa^H1c-GFP^) and U2OS cells stably expressing GFP-tagged HMGB1 and RFP-tagged H2B (U2OS^2FP^), (from the labs of Profs. Hiroshi Kimura, Tokyo and Guido Kroemer, Paris, respectively) were maintained in DMEM medium (Gibco) supplemented with 10% FBS 1 × GlutaMAX, 100 U/ml penicillin, 100 µl/ml streptomycin and phenol red. In addition, media for U2OS^2FP^ cells was supplemented with G418 0.5 mg/mL for continuous selection of transformed cells. HeLa cells and HeLa cells stably expressing EGFP-RARα, (HeLa^EGFP-RARα^) were maintained in RPMI supplemented with phenol red, 10% fetal calf serum (Sigma-Aldrich, Saint Louis, MO), 1 × GlutaMAX (Fisher Scientific, Tokyo, Japan), and 50 mg/l gentamycin (KARA, Novo Mesto, Slovenia). All cells were cultured at 37 °C in a humidified, 5% CO_2_ incubator and passaged every two days. The expression of the tagged proteins in the transfected cell lines was ~ 20% of the levels of the respective native proteins in the case of H1c-GFP and HMGB1-GFP (data not shown), while the expression level of transfected EGFP-RARα was double of the endogenous level of RARα as determined by RT-QPCR^18^.

For microscopy experiments, HeLa or HeLa^EGFP-RARα^ cells were seeded in 8-well chambered cover slips (ibidi GmbH, Gräfelfing, Germany) 48 h before the measurement and maintained in phenol red-free RPMI supplemented with 10% charcoal-stripped fetal calf serum (PAN-Biotech, Aidenbach, Germany).

### Transfection

Twenty-four hour after seeding, HeLa cells reached 50–60% confluency and were transiently transfected with 80 ng of EGFP dimer for FRAP experiment and 40 ng for the FCS experiments using FuGENE^®^ HD transfection reagent (Promega, MA, USA) as suggested by the manufacturer.

### Cell treatment

Cells were treated with the indicated concentrations of either Dox for 2 h (Sigma Aldrich), EBr for 1 h, bleomycin for 2 h, Hoechst 33,342 (10 µM) for 30 min or H_2_O_2_ for 20 min at 37 °C in phenol red-free medium, in a volume of 300 µl/well. Irradiation with X-rays was done at room temperature at a distance of 57.6 cm from the window of a 6 MeV linear accelerator (Radiotherapy Department, University of Debrecen) while the cells were kept in phenol red-free cell culture medium (300 µl/well). AM580 was applied to the cells at 0.1 μM final concentration for 30 min at 37 °C before imaging.

### Point FRAP

When it was made possible by the kinetics of recovery, point FRAP was used to better detect possible spatial heterogeneities. For measurement of GFP-HMGB1 mobility, point FRAP measurements were performed on an Olympus FluoView 1000 confocal microscope based on an inverted IX-81 stand with an UPlanAPo 60 × NA 1.2 oil immersion objective. EGFP was excited by the 488 nm line of an Ar-ion laser, and emission was detected through a 500–520 nm band-pass filter by a PMT. The measurement for point FRAP data acquisition started with a confocal image of a cell (512 × 512-pixel, pixel size: 0.103 µm), followed by the selection of a laser spot at which the laser beam was focused. Before bleaching, 5120 pre-bleach pixels were collected with a pixel dwell time of 10 μs (51.2 ms) followed by bleach period for 51.2 ms with 100% laser power 55.6 µW and then collecting 40,000 post-bleach pixels from the same spot for a total time of 297.59 ms.

In order to change the laser power of the Ar-ion laser shorter than the pixel dwell time, a dedicated LabVIEW program was developed. The analogue output of a NI 7833 field programmable gate array (FPGA) card (National Instruments, Austin, TX) was fed into the laser power controller input pin of the acousto-optic tunable filter (AOTF, AA Opto electronic MOD.NC) of the laser combiner unit, allowing fast (~ 1 μs) voltage driven laser power switching by an FPGA card. The operating of the FPGA card was initiated by a TTL output of the trigger port of the FV1000 system, thus controlling the laser power and collecting the fluorescence data were synchronized.

The model function fitting of the recovery post-bleach data was performed by a custom-written Matlab (The Math Works, Natick, MA) program. Data were fitted assuming a one-component exponential recovery with the following equation:

1 $$I\left( t \right) = I_{0} \left( {1 - e^{{ - {\raise0.7ex\hbox{$t$} \!\mathord{\left/ {\vphantom {t \tau }}\right.\kern-\nulldelimiterspace} \!\lower0.7ex\hbox{$\tau $}}}} } \right) + I_{bg}$$

where *I*_(t)_ is intensity at a given time point; t, time; *I*_0_, amplitude of the intensity; τ, recovery time; *I*_bg_, background intensity.

### Strip FRAP

When point FRAP recoveries were too fast to provide accurate mobility data, a strip FRAP protocol was followed. These FRAP measurements for histone H1-GFP were carried out on an Olympus FluoView 1000 confocal microscope as described previously^17^ with a minor modification; 10 images (512 × 512 pixels, 4 µs/pixel), were collected before bleaching, followed by 15 bleaching images in a ROI (25 × 12 pixels, 20 µs/pixel, 56 µW) and 80 post-bleach images.

FRAP measurements for EGFP-RARα and EGFP dimers were also carried out in a similar setting with some modifications. In brief, measurements were performed on a Zeiss LSM 880 (Carl Zeiss, Jena, Germany) confocal microscope using a 40 ×, 1.2 NA water immersion objective. The 488 nm laser line was used to excite EGFP with a laser power of 2 μW at the objective (10%), and emission was detected through a 493 to 529 nm band pass filter. For quantitative analysis, a 256 × 256-pixel area was selected and scanned with an open pinhole (5.56 Airy units) and 10 × zoom (pixel size: 0.08 µm), with a pixel dwell time of 1.33 μs. A 405-nm laser was used to bleach the EGFP molecules at a selected strip-shaped region of interest (FRAP ROI) having an area of 140 × 10 pixels, a laser power of 20 μW at the objective, and a pixel dwell time of 8.24 μs. Before bleaching, 10 images were collected at a repetition rate of 204.8 ms/frame followed by one bleach period at the FRAP ROI, and then collecting 189 post-bleach images for a total time of 42 s. To standardize the geometry of the measurement, the scanned field was rotated to make the long axis of the selected nucleus vertically oriented in the image, and the strip shaped FRAP ROI (bleached area) was positioned horizontally at one third of the vertical extension of the nucleus, avoiding nucleoli (Fig. S12).

Images were processed using the open-source FIJI distribution of ImageJ (version 2.0.0-rc-69/1.52i) to acquire the fluorescence intensity recovery curves required for FRAP analysis. The width of the FRAP ROI was cropped to match the width of the nucleus. Another ROI contouring the whole nucleus but excluding the nucleoli was made to calculate the total fluorescence intensity of the nucleus. A third ROI outside the cell was drawn to calculate the intensity of the background. Following the double normalization method^54^ the intensity in the FRAP ROI during the recovery period was normalized to its pre-bleach value *I*_*ROI*_*(0)*, and corrected for acquisition bleaching of the whole nucleus using the following equation:

2 $$I_{norm} \left( t \right) = \frac{{I_{ROI} \left( t \right) - I_{B} }}{{I_{ROI} \left( 0 \right) - I_{B} }} \times \frac{{I_{total} \left( 0 \right) - I_{B} }}{{I_{total} \left( t \right) - I_{B} }}$$

where *I*_*ROI*_*(t)* is intensity of the FRAP ROI at a given time during the recovery, *I*_*ROI*_*(0)* is average intensity of the ROI before bleaching, *I*_*total*_*(t)* is the intensity of the whole nucleus at a given time during recovery, *I*_*total*_*(0)* is the average intensity of the whole nucleus before bleaching over ten frames, and *I*_*B*_ is average intensity of the background. Using the Prism version 8.4.0 software, a two-component exponential curve (Eq. 3) was fitted to the normalized recovery curve of the EGFP-RAR while a one-component exponential to the EGFP dimer:

3 $$I_{norm} \left( t \right) = \left( {I_{\min } - I_{\infty } } \right) \times \left( {r_{fast} \exp \left( {{{ - t} \mathord{\left/ {\vphantom {{ - t} {\tau_{fast} }}} \right. \kern-\nulldelimiterspace} {\tau_{fast} }}} \right) + r_{slow} \exp \left( { - {t \mathord{\left/ {\vphantom {t {\tau_{slow} }}} \right. \kern-\nulldelimiterspace} {\tau_{slow} }}} \right)} \right) + I_{\infty }$$

The fit yielded the *τ*_*fast*_ and *τ*_*slow*_ recovery times of the fast and the slow components, their fractions *r*_*fast*_ and *r*_*slow*_ adding up to 1, the plateau *I*_*∞*_ at infinite time and the fitted intensity value right after bleaching, *I*_*min*_. The mobile fraction was determined as:

4 $$r_{mobile} = {{\left( {I_{\infty } - I_{\min } } \right)} \mathord{\left/ {\vphantom {{\left( {I_{\infty } - I_{\min } } \right)} {\left( {1 - I_{\min } } \right)}}} \right. \kern-\nulldelimiterspace} {\left( {1 - I_{\min } } \right)}}$$

The average recovery time was calculated as a weighted average of the fast and slow components:

5 $$\tau_{average} = r_{fast} \tau_{fast} + r_{slow} \tau_{slow}$$

### Fluorescence correlation spectroscopy (FCS)

FCS measurements were carried out in 300 µl volume on an 8-well chamber at room temperature. For the in vitro setting, 1 µg of native pEGFP-C3 plasmid DNA and 945 ng of Alexa 647 labelled rHMGB1 were mixed in protein binding buffer with or without doxorubicin. The mixture was allowed to equilibrate at RT for 1 h before measurement. For the in vivo setting, HeLa cells expressing EGFP-RAR or EGFP dimer, treated with 0 or 200 µM H_2_O_2_ were examined for a maximal duration of 20 min.

FCS measurements were carried out on a Nikon A1 Eclipse Ti2 confocal laser-scanning microscope (Nikon, Tokyo, Japan), equipped with a Plan Apo 60 × water immersion objective [NA = 1.27] and a PicoQuant-TCSPC-FCS upgrade kit (PicoQuant, Berlin, Germany). EGFP and Alexa 647 were excited by the 488 nm and 633 nm lasers, respectively. The fluorescence emission was filtered through 488–546 nm and 650–700 nm band width filters and detected with single photon counting detectors (PicoQuant, Berlin, Germany). Measurements of 10 × 8 s runs were taken at a point in the solution/cell. Fluorescence autocorrelation curves were calculated with the SymPhoTime64 software (PicoQuant, Berlin, Germany) at 200 time points from 300 ns to 1 s with quasi-logarithmic time scale. Autocorrelation curves were fitted to a model with triplet state and two diffusion components to account for DNA-bound (slow component) and freely diffusing (fast component) proteins.

6 $$G\left( \tau \right) = \frac{{1 - T + Te^{{ - \frac{\tau }{{\tau_{tr} }}}} }}{{N\left( {1 - T} \right)}} \left( {\rho \frac{1}{{1 + \frac{\tau }{{\tau_{D1} }}}}\frac{1}{{\sqrt {1 + \frac{\tau }{{S^{2} \tau_{D1} }}} }} + \left( {1 - \rho } \right)\frac{1}{{\sqrt {1 + \frac{\tau }{{\tau_{D2} }}} }}\frac{1}{{\sqrt {1 + \frac{\tau }{{S^{2} \tau_{D2} }}} }}} \right)$$

where *N* is the average number of fluorescent molecules in the detection volume, *T* is the fraction of molecules in the triplet state, *τ*_*tr*_ is the triplet correlation time. The rate of diffusion is characterized by the diffusion time, *τ*_*d*_, which is the average time that a molecule spends in the illuminated volume. *τ*_*D1*_ and *τ*_*D2*_ are the diffusion times of the fast and slow components, *ρ* is the fraction of the first component and 1−ρ is the fraction of the second component. Diffusion coefficients (*D*) of the fast and slow components were determined from the following equation:

7 $$D = \frac{{\omega_{xy}^{2} }}{{4\tau_{D} }}$$

where *ω*_*xy*_ is the lateral e^−2^ radius of the detection volume. *ω*_*xy*_ was measured by determining the diffusion time of 100 nM Alexa 488 (for calibrating EGFP-RARα diffusion) or Alexa 647 dye (dissolved in 10 mM Tris–EDTA buffer, pH 7.4) with a known diffusion coefficient (*D*_A647_ = 309.1 µm^2^/s^55,56^, *D*_A488_ = 435 µm^2^/s^57^ at T = 22.5 °C) and substituting them into Eq. 3. All correlation curves were fitted using the free QuickFit 3.0 software^57^. FRAP and FCS data were plotted using GraphPad Prism version 8.0.2.

### Expression and purification of rHMGB1

The pET19b expression vector carrying the full length human HMGB1 gene with C23S, C45S, C106S and E204C mutations (from Jennifer Kugel’s lab, University of Colorado Boulder, USA) was transformed into Rossetta (DE3)pLysS and grown overnight at 37 °C incubator on LB agar plates containing 100 µg/ml ampicillin and 37.4 µg/ml chloramphenicol. A single transformed colony from the plate was used to inoculate 5 ml LB with antibiotic. The small culture was incubated overnight at 37 °C with shaking and was used to inoculate 100 ml of LB containing D glucose (100 ml LB, 2.4 mM NaOH, 0.2 g D-glucose) and antibiotic in a 500 ml culture flask. The culture was grown at 37 °C with shaking until an OD600 of 0.5 was attained upon which expression was induced using 0.5 mM IPTG and the cells were grown for a further 3 h. The culture was transferred into 50 ml centrifuge tubes and pelleted at 5000 RPM for 15 min. The supernatant was discarded leaving a small volume to resuspend the pellet in. The cells were then transferred into a 1.5 ml Eppendorf tube and pelleted again. The supernatant was discarded, the pellet flash-frozen and stored at − 80 °C.

To extract the protein, 2 ml of lysis buffer (20 mM Tris pH 7.9, 500 ml NaCl, 10% glycerol, 5 mM imidazole, 5 mM beta mercaptoethanol (BME) and 0.2 mM PMSF) was added to a thawed pellet from 100 ml culture, the pellet was resuspended then the cells sonicated 5 × on ice (30 s on, 30 s off). The lysate was then centrifuged for 15 min at 4 °C at 15,000 RPM. The supernatant containing the protein was transferred into a packed Nickel column and then kept at 4 °C for 1 h with rocking motion. The supernatant was allowed to flow through, then the resin washed with 1 ml of lysis buffer followed by 3 ml of lysis buffer containing 50 mM imidazole. The protein was eluted using lysis buffer containing 250 mM imidazole. The column was stripped with lysis buffer supplemented with 1 M imidazole.

Following elution from the nickel column, the buffer was exchanged using a 10 K MWCO spin column and 50 ml degassed dialysis buffer (20 mM Tris pH 7.9, 50 mM KCL, 10% glycerol, 100 mM PMSF, 100 mM MgCl_2_ and 1 mM DTT). After buffer exchange the protein sample in about 250 µl was centrifuged at 18,000 RPM for 30 min at 4 °C and the supernatant transferred to a dsDNA cellulose column and incubated for 1 h at 4 °C with rocking motion. The supernatant was allowed to flow through, the resin washed 3 × with 3 ml wash buffer (20 mM Tris pH 7.9, 50 mM NaCl, 10% glycerol, 5 mM MgCl_2_ and 1 mM DTT) and the protein eluted using wash buffer with 500 mM NaCl. The column was stripped with wash buffer containing 1 M KCl. The protein was then desalted using 10 K MWCO spin column and degassed dialysis buffer as above without DTT. Purification was confirmed using a 12% SDS PAGE gel electrophoresed for 1 h at 150 V (Fig. S3a). Samples were centrifuged at 18,000 RPM for 30 min at 4 °C, aliquoted, flash frozen and stored at − 80 °C. Quantification was carried out using Lowry assay and known amounts of BSA controls. Measurements were also made on the Nanodrop.

### Immunofluorescence labelling of rHMGB1

Labelling of rHMGB1 with Alexa 647 C2 maleimide (Thermo scientific) was done following the manufacturer’s instructions. To reduce the disulphide bonds in the protein, 750 µg of rHMGB1 in degassed buffer A (100 mM Tris pH 7.1, 50 mM KCl, 10% glycerol, 0.2 mM PMSF and 5 mM MgCl_2_) was mixed with DTT to a final concentration of 10 mM and incubated at 4 °C for 2 h. DTT was removed using a spin column and 20 ml buffer A. 1.5 µl of 8 mM Alexa 647 C2 maleimide was added to the sample and incubated in the dark at 4 °C overnight. Unbound dye was removed using 10 K MWCO microfuge spin column and sample washed with column wash buffer (20 mM Tris pH 7.9, 50 mM KCl and 5 mM MgCl_2_). Samples were quantified by measuring absorbance at 280 nm and compared to a standard curve prepared from known amounts of BSA. Glycerol was added to a final concentration of 10% and samples stored at either − 20 °C for short term or − 80 °C for long-term storage.

### Electrophoretic mobility shift assay (EMSA)

To determine the binding affinity of the labelled rHMGB1 to various topological forms of plasmid DNA, gel electrophoresis was carried out. Native pEGFP C3 plasmid DNA was either nicked or linearized using Nb.Mva1269I and ECoRI (Thermo scientific), respectively, using the manufacturer’s instructions. The DNA samples were then cleaned using gel cleaning kit (QIAGEN) following the manufacturer’s instructions. DNA was quantified by measuring the absorbance at 260 nm on the Nanodrop. An equal amount (0.5 µg) of linear, nicked and native plasmid DNA was mixed in 20 µl of protein binding buffer (50 mM NaCl, 20 mM Tris HCl pH7.5 and 0.2 mM EDTA). To this DNA mixture, varying amounts of rHMGB1 was added per sample as indicated on the gel (Fig. S3) and incubated on ice for 40 min on ice. The samples were then loaded to a 1% agarose gel in 0.5 × TBE and run for at 36 V for 15 h at 4 °C. The gel was stained with 0.5 µg/ml EBr and then imaged. The image was processed using Fiji ImageJ.

### Statistical analysis

For creating plots and for statistical comparisons GraphPad Prism 8.01 was used. Box-and-whiskers plots represent 10th, 25th, 50th, 75th, and 90th percentiles; + , mean value. To compare averages of multiple data sets, ANOVA with Tukey’s multiple comparison test was used.

## Supplementary Information

Supplementary Information.

## Data Availability

All data will be made available upon request. Please contact Gábor Szabó (szabog@med.unideb.hu) or György Vámosi (vamosig@med.unideb.hu).
