# Supplementary material for: Doxorubicin impacts chromatin binding of HMGB1, Histone H1 and retinoic acid receptor
Source: Sci Rep. 2022 May 16;12:8087. doi: 10.1038/s41598-022-11994-z (PMC9110345; doi:10.1038/s41598-022-11994-z)
Supplement: Supplementary file 1 — Supplementary Information. [file 41598_2022_11994_MOESM1_ESM.pdf]

Supplemental material for

# DOXORUBICIN IMPACTS CHROMATIN BINDING OF HMGB1, HISTONE H1 AND RETINOIC ACID RECEPTOR

Rosevalentine Bosire[1,2], Lina Fadel[1,3], Gábor Mocsár[1], Péter Nánási jr.[1], Pialy Sen[1,3], Anshu Kumar Sharma[1], Muhammad Umair Naseem[1,3], Attila Kovács[4], Jennifer Kugel[5], Guido Kroemer[6,7,8,9,10], György Vámosi[1*] & Gábor Szabó[1*]

[1]Department of Biophysics and Cell Biology, Faculty of Medicine, University of Debrecen, Debrecen, Hungary
2Doctoral School of Molecular Cell and Immune Biology, University of Debrecen, Debrecen, [3]Doctoral School of Molecular Medicine, University of Debrecen, Debrecen, Hungary
Hungary
[4]Department of Radiation Therapy, Faculty of Medicine, University of Debrecen, Debrecen, Hungary
[5]Department of Biochemistry, University of Colorado, Boulder
[6]Centre de Recherche des Cordeliers, Equipe labellisée par la Ligue contre le cancer, Université de Paris, Sorbonne Université, Paris, France
[7]Metabolomics and Cell Biology Platforms, Institut Gustave Roussy, Villejuif, France
[8]Pôle de Biologie, Hôpital Européen Georges Pompidou, Paris, AP-HP, France
[9]Suzhou Institute for Systems Medicine, Chinese Academy of Medical Sciences, Suzhou, China. [10]Karolinska Institute, Department of Women's and Children's Health, Karolinska University Hospital, Hospital

*Equal senior, corresponding authors: szabog@med.unideb.hu, vamosig@med.unideb.hu

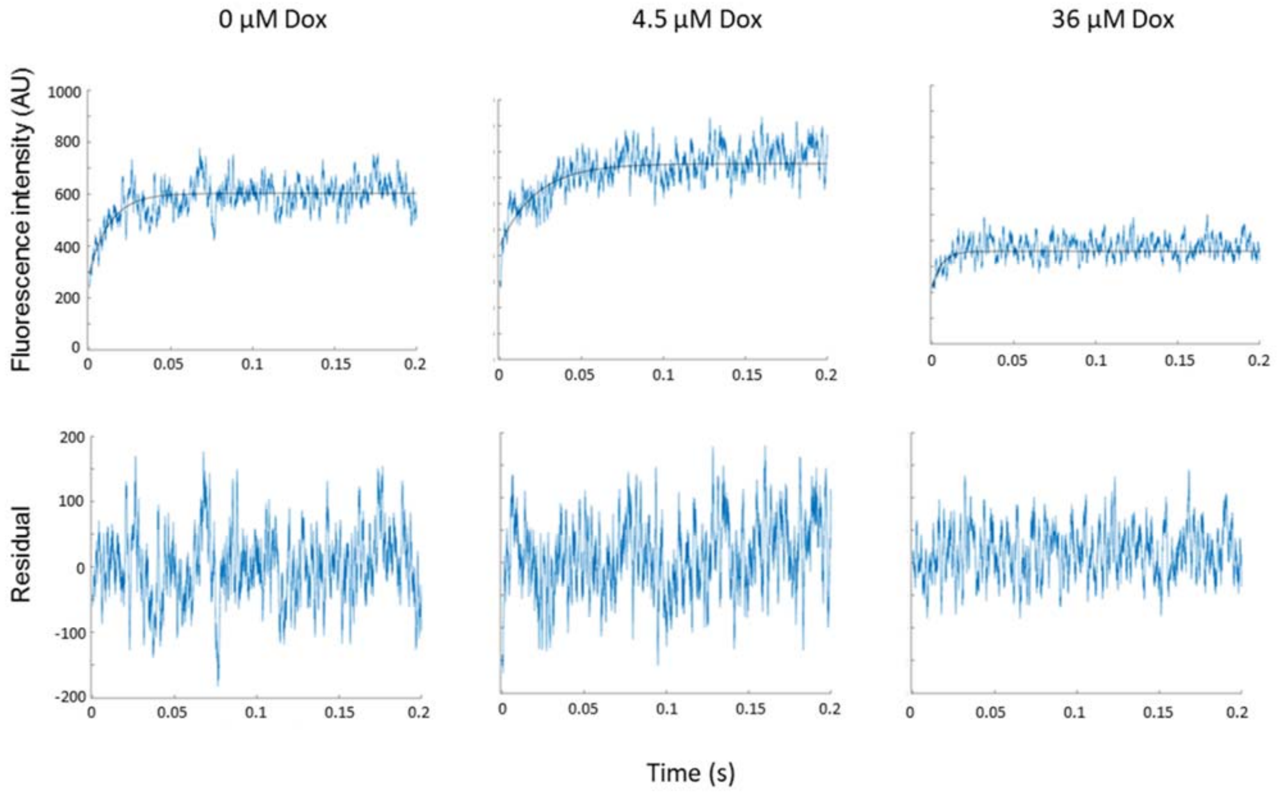


**Figure S1: Doxorubicin affects HMGB1 dynamics in a concentration dependent manner**

Representative point FRAP curves of GFP-HMGB1 recorded in nuclei of U2OS2FP cells treated with the indicated concentrations of Dox for 2 hrs. Top: raw fluorescence recovery curves fitted to monoexponential decays. Bottom: fit residuals.

**a**

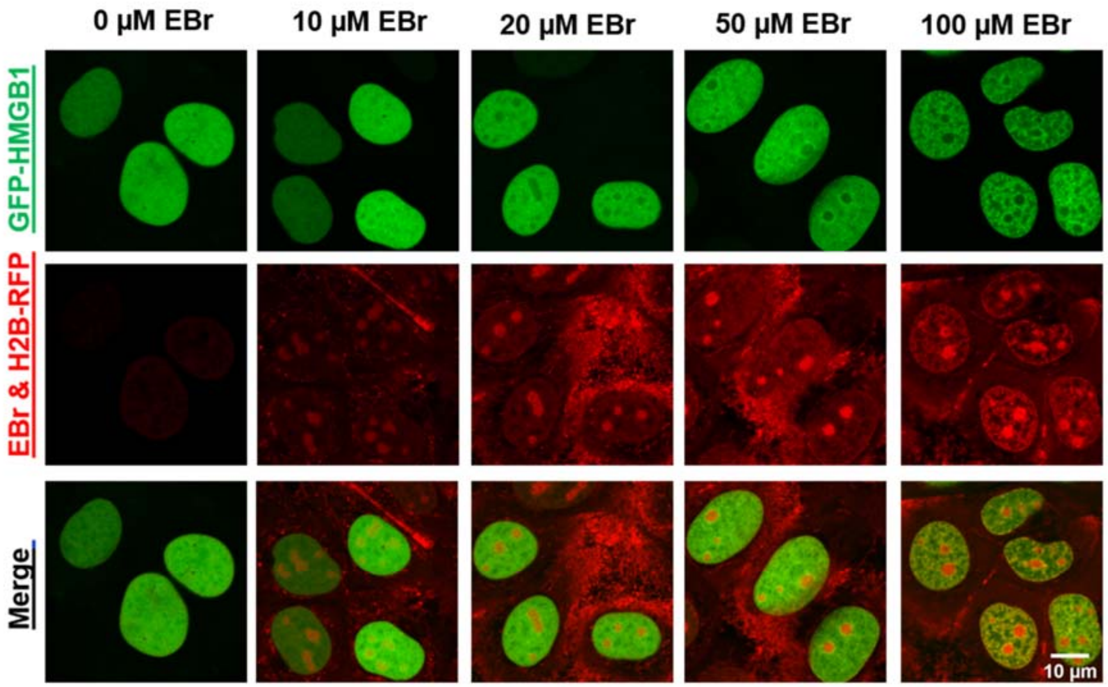


**b**

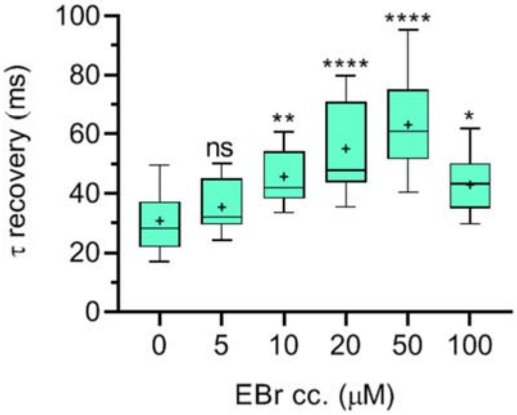


**Figure S2: EBr causes redistribution of HMGB1 within the nuclei and exerts a biphasic effect on HMGB1 mobility in live cells** U2OS[2FP] cells were incubated with 0, 10, 20, 50 and 100 μM EBr for 1 hr and then imaged by confocal microscopy. **a**) Representative nuclei showing GFP-HMGB1, EBr and H2B-RFP and the merged images. Uptake of EBr is evident from the appearance of nucleolar and cytoplasmic fluorescence, as well as increased chromatin fluorescence. At 100 μM EBr, chromatin condensation can also be observed. H2B-RFP hardly contributes to the red signal, therefore the fluorescence gain of that channel in the control sample (0 EBr) was increased on the image to make H2B-RFP visible. Following EBr treatment, there is gradual loss of GFP from the nucleoli and its distribution in chromatin becomes more structured **b**) Fluorescence recovery time of GFP-HMGB1 in EBr treated cells as measured by point FRAP. Following treatment with EBr, GFP-HMGB1 recovery time increases peaking at 50 μM. One-way ANOVA with post hoc Dunnett's test was used to calculate significance of differences relative to 0 EBr. *$p < 0.05$, **$p<0.01$, ****$p<0.0001$.

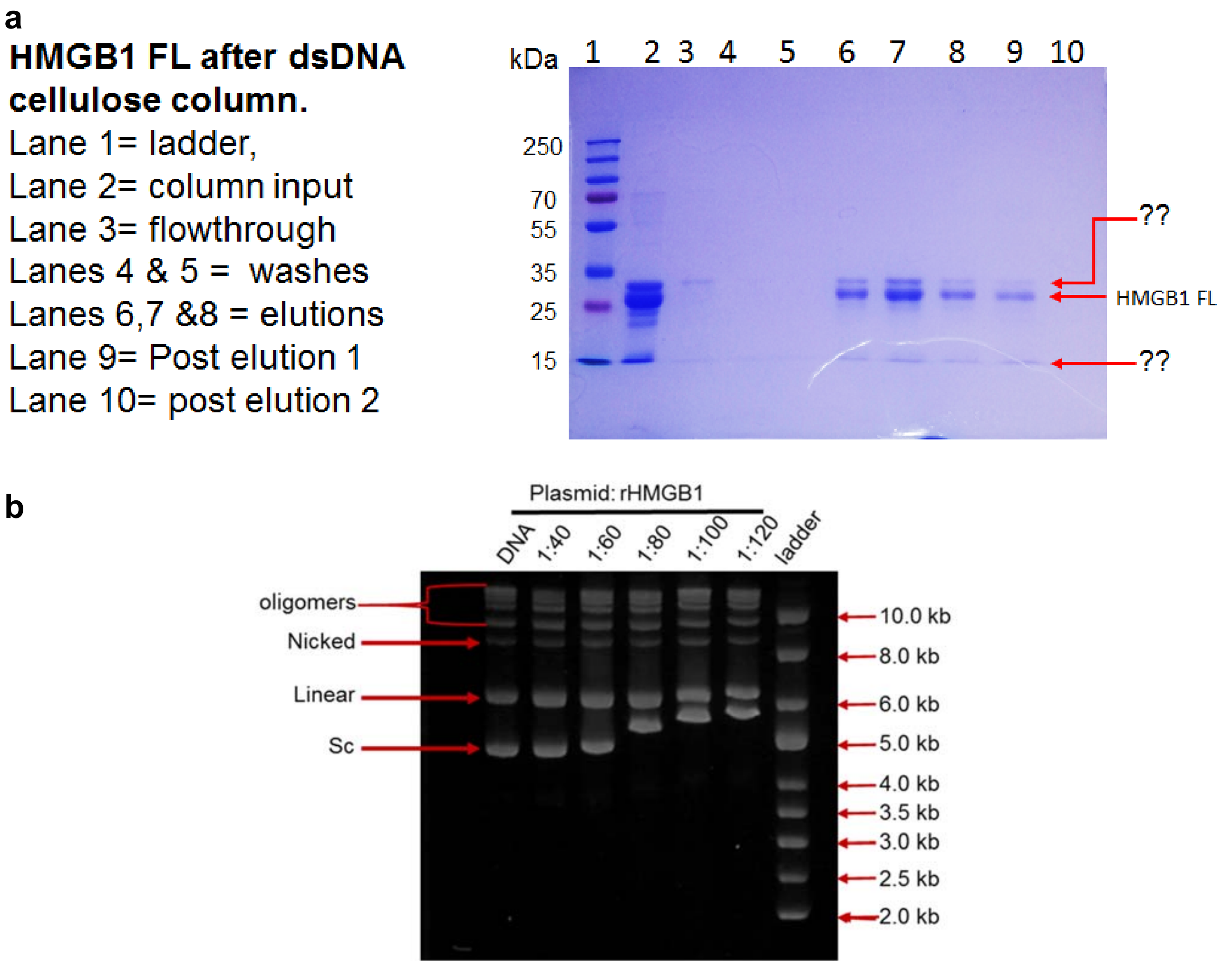


**Figure S3: (a)** SDS PAGE analyses of the rHMGB1 protein used in our experiments. The purity was at least 70%. The extra bands did not affect the FRET measurements performed with a similar preparation[1]. **(b)** Varying amounts of rHMGB1 were added and allowed to bind 1.5 µg of plasmid DNA containing nicked, linear and supercoiled forms in equal amounts, before being separated by gel elecrophoresis. The gel was stained with 0.5 µg/ml EBr following electrophoresis. Below, the unprocessed gel photo is shown.

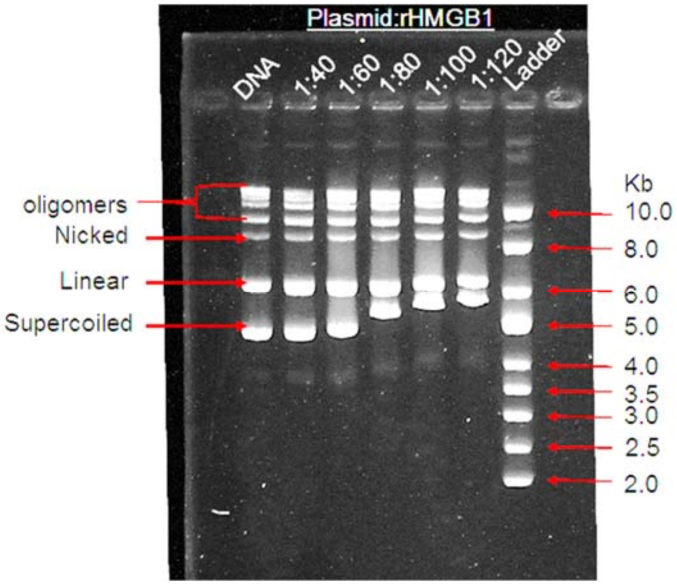


Unprocessed gel photo

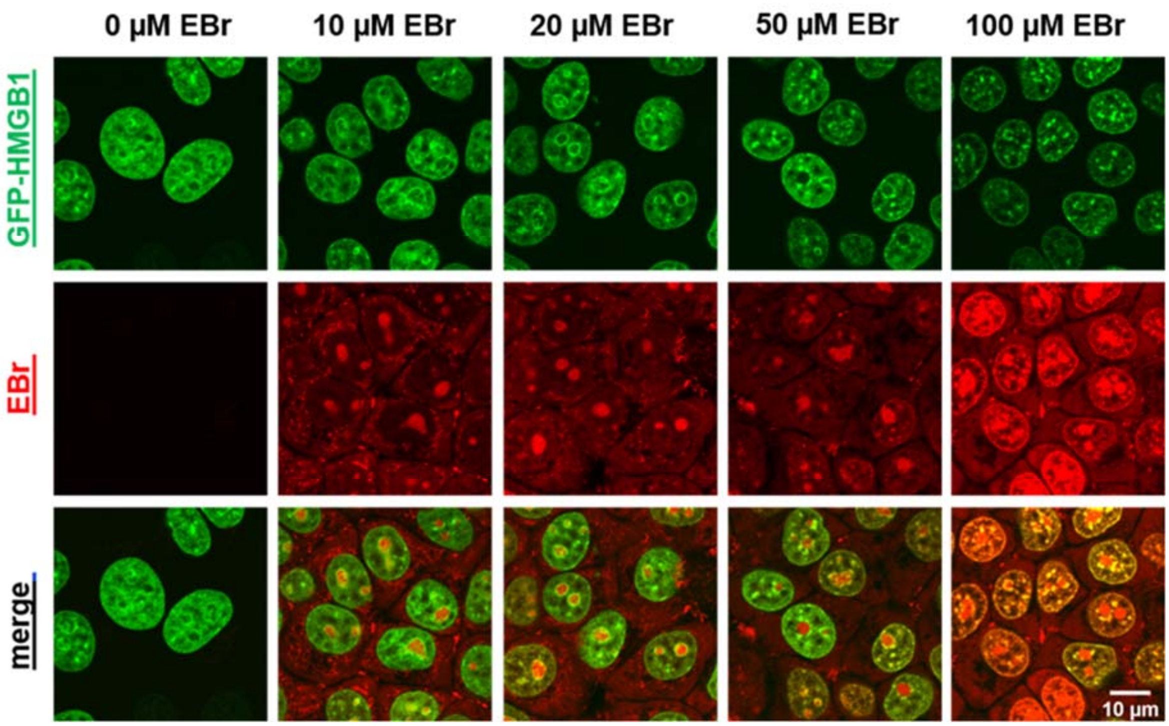


**Figure S4: EBr displaces histone H1c from chromatin**

Live HeLa cells expressing GFP tagged histone H1c were treated with varying concentrations of EBr for 1 hr. Representative nuclei from EBr treated cells are shown.

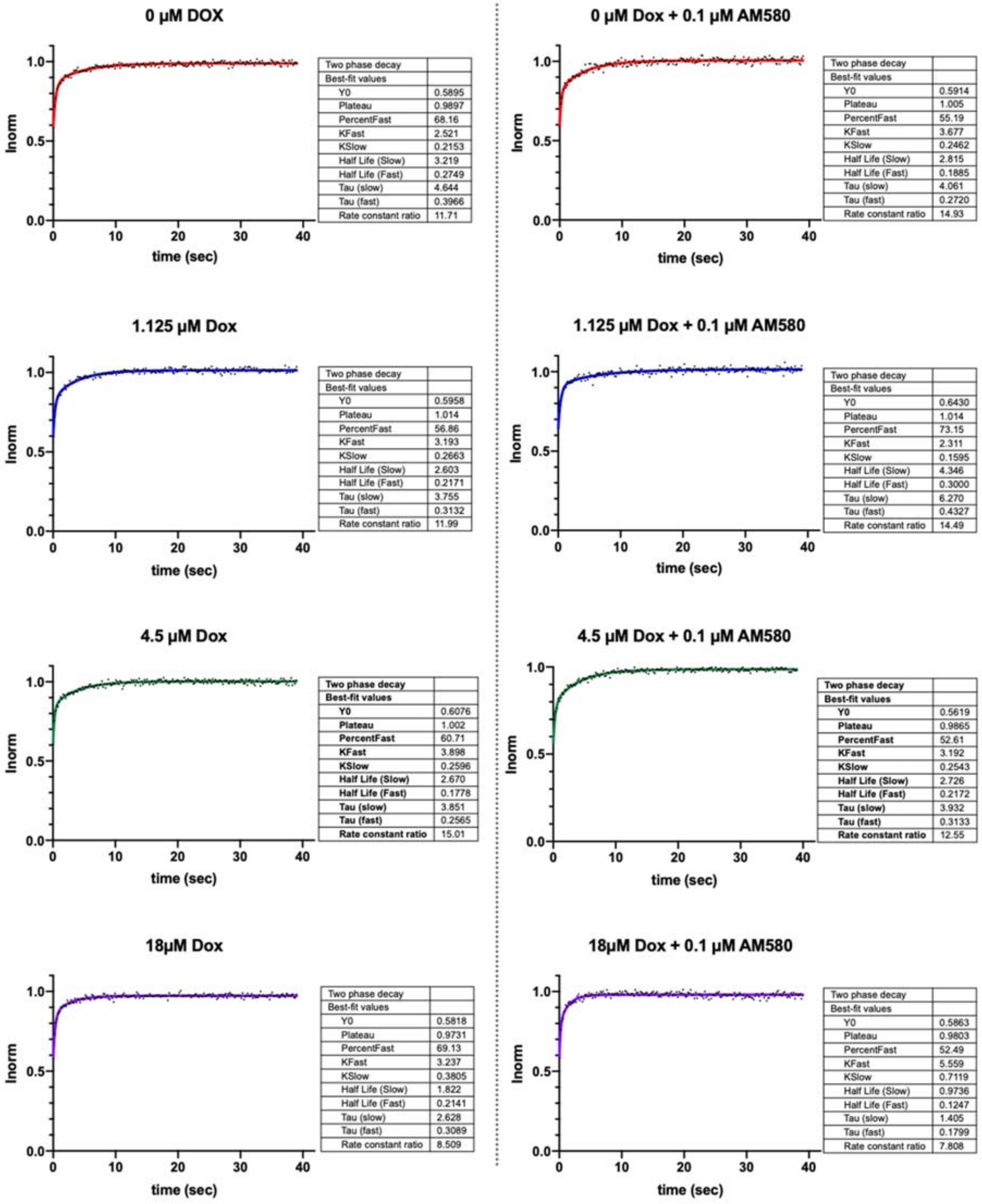


**Figure S5: Representative two-component exponential fits of EGFP-RARα**

Normalized intensities and fits using GraphPad Prism version 8.4.0 are shown. The curves represent EGFP-RARα normalized intensities at different concentrations of doxorubicin (0, 1.125, 4.5, 18 μM) in the presence or absence of RARα specific agonist treatment (0.1 μM AM580).

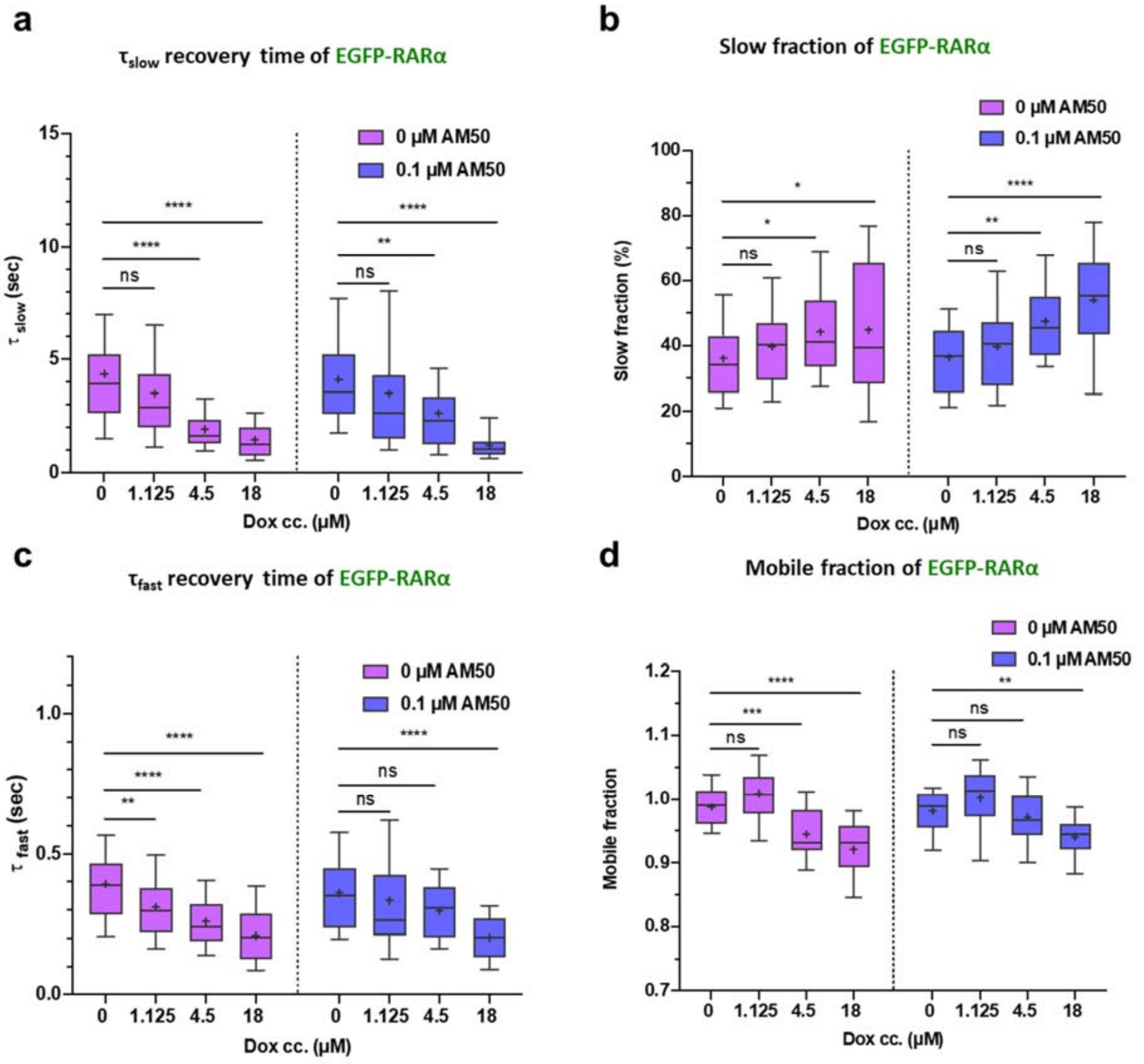


**Figure S6: Recovery times and fractions of the slow and fast components of EGFP-RARα expressed in HeLa cells, determined in strip FRAP experiments**

(**a**) Recovery times of the slow component in cells treated with the indicated concentrations of Dox and/or RAR agonist AM580 for 1 hr; (**b**) fractions of the slow component; (**c**) recovery times of the fast component; (**d**) fraction of the mobile component. In the figures, the p values are defined by Tukey's multiple comparison test. * $p<0.5$; ** $p<0.1$; *** $p<0.01$; **** $p<0.001$; ns, not significant.

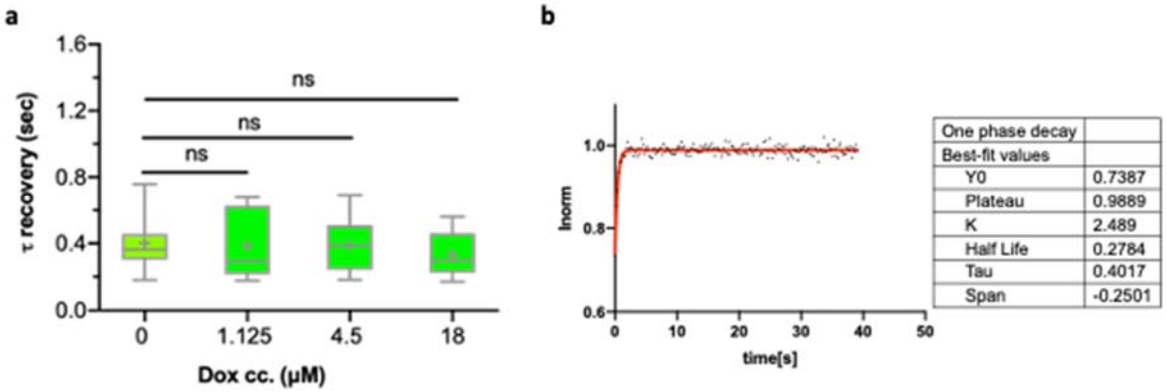


**Figure S7: Nuclear mobility of EGFP dimer is unaffected by Dox treatment**

Recovery time of an inert protein, EGFP dimer as a control for FRAP experiments. (**a**) $\tau_{average}$ recovery times for the EGFP dimer at different concentrations of doxorubicin (0, 1.125, 4.5, 18 μM). (**b**) Representative one-component exponential decay fit of the normalized intensity of the EGFP dimer. One-way ANOVA with post hoc Dunnett's test was used to calculate significance; $p<0.05$ was considered a significant change; ns: not significant.

**Supplementary Discussion to Fig. S7**

Dox has previously been shown to alter the overall chromatin structure through core histone eviction and histone aggregation [2,3]. Such an altered environment may affect the microviscosity of the nucleus allowing for faster diffusion. To learn if reduced FRAP recovery times of RARα (Fig. 5) reflect reduced binding or decreased viscosity, the effect of Dox on diffusion of EGFP dimers, having no known binding sites on chromatin, was measured. Dox had no effect on its recovery time (Fig. S7a) indicating that the average microviscosity in the nucleus did not change to an extent that would influence the diffusion of proteins of this size (EGFP dimer: ~54 kDa, EGFP-RAR: 78 kDa) in the nucleus. Thus, the reduced FRAP recovery time of RARα indeed reflects reduced binding. We also measured the local mobility of EGFP dimers by FCS. The FCS-derived D value displayed no significant change upon 4.5 μM Dox treatment either (Fig. S8c).

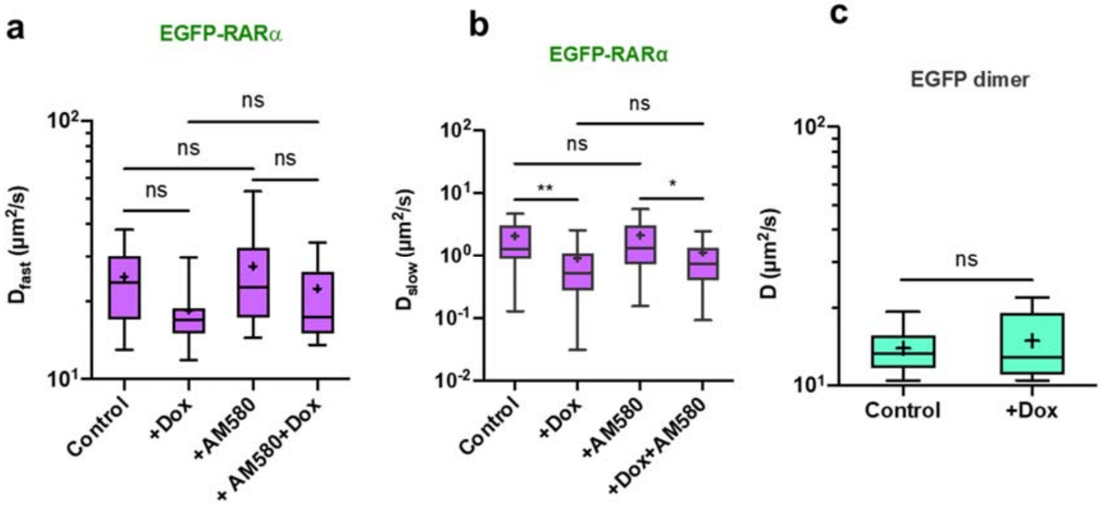


**Figure S8: Diffusion constants of EGFP-RARα and EGFP dimer**

Diffusion constants of **a)** the fast, freely diffusing or transiently DNA-bound component of EGFP-RARα, and **b)** the slow, DNA-bound component in control and Dox-treated (4.5 µM) HeLa cells stably expressing the receptor. The increased variability of $D_{slow}$ in Dox-treated cells may reflect the more heterogeneous distribution of GFP-RARα as shown in Fig. 6. **c)** The diffusion constant of the EGFP dimer did not change significantly following 4.5 µM Dox treatment. Two-way ANOVA with Tukey's multiple comparison test was used to calculate significance. * $p<0.05$; ** $p<0.01$; ns, not significant.

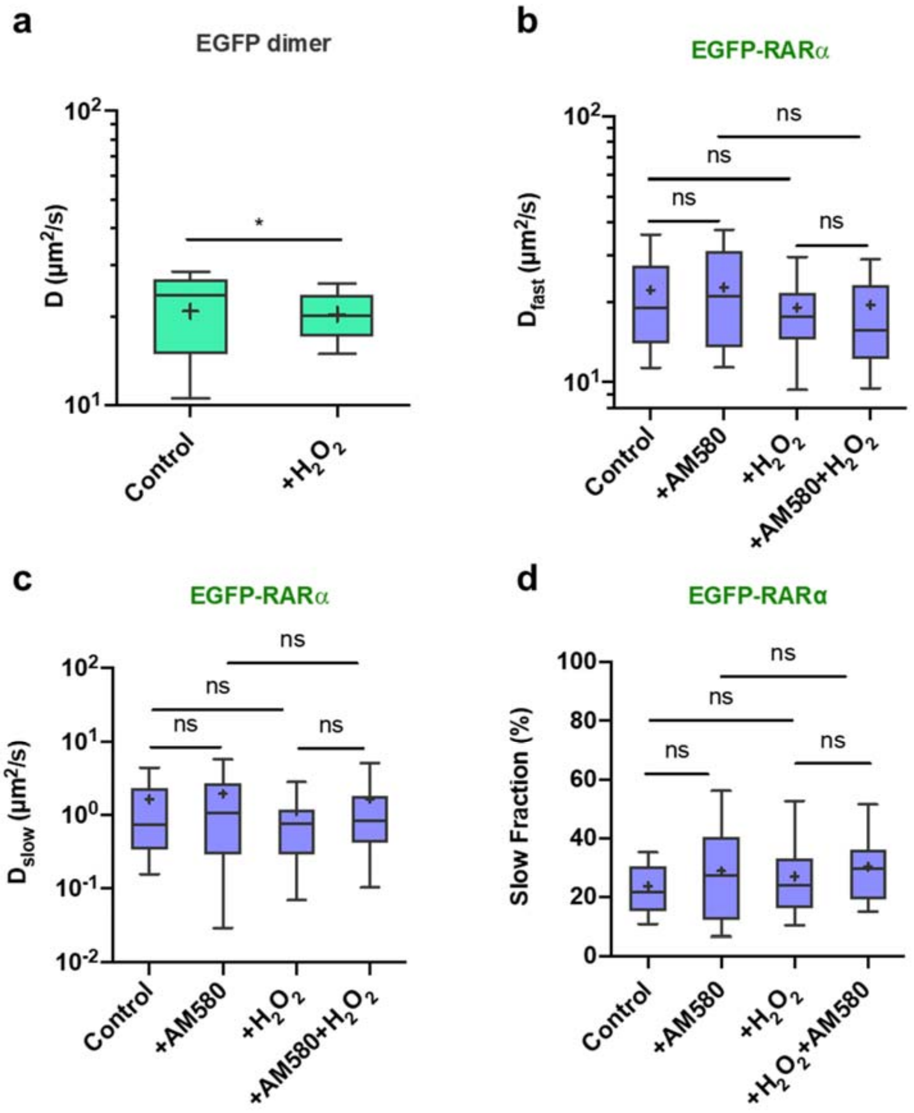


**Figure S9: $H_2O_2$ has no effect on DNA-binding and diffusion of RARα in HeLa cells**

Diffusion properties of EGFP-RARα and the inert protein EGFP dimer as a control were measured by FCS. $H_2O_2$ was used at 200 µM while AM580 concentration was 0.1 µM (**a**) Diffusion coefficient of EGFP dimer upon $H_2O_2$ treatment. (**b**) Diffusion coefficient of freely diffusing EGFP-RARα (fast component) and (**c**) DNA-bound EGFP-RARα (slow component). (**d**) Fraction of DNA-bound EGFP-RARα (slow component). p values are defined by Tukey's multiple comparison test where, *($p<0.5$), **($p<0.1$), ***($p<0.01$), ****($p<0.001$), ns, not significant.

**a** EGFP dimer

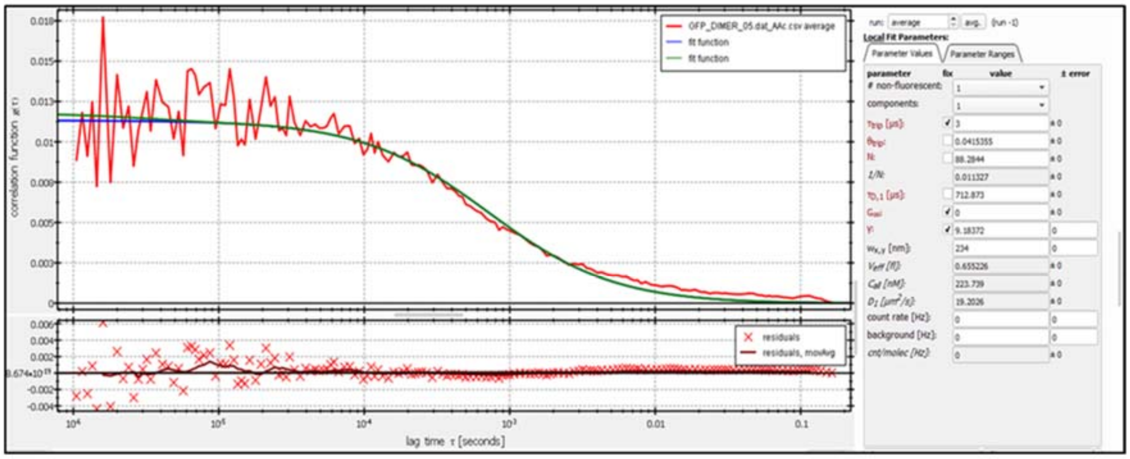


**b** EGFP dimer + $H_2O_2$

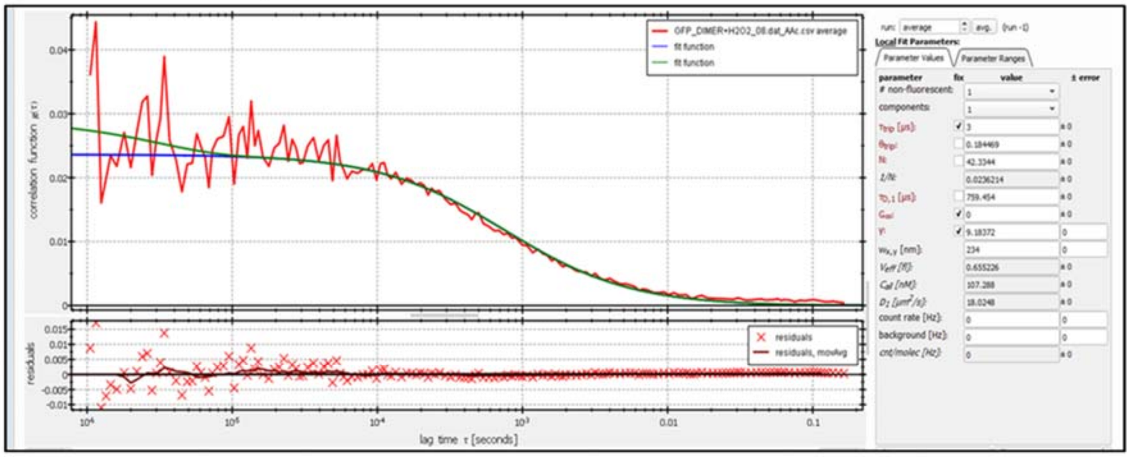


**c** EGFP-RARα + AM580

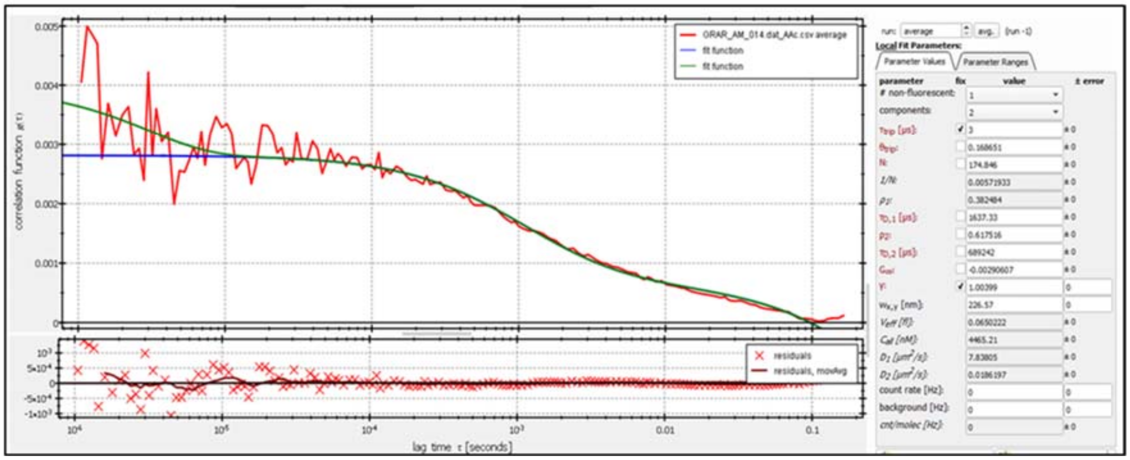

**d** EGFP-RARα + AM580 + $H_2O_2$

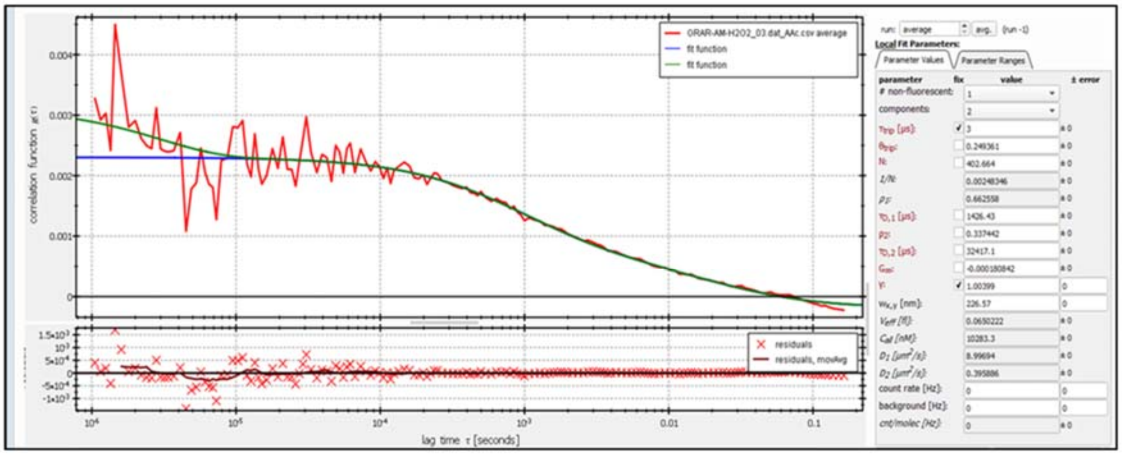


**e** EGFP-RARα

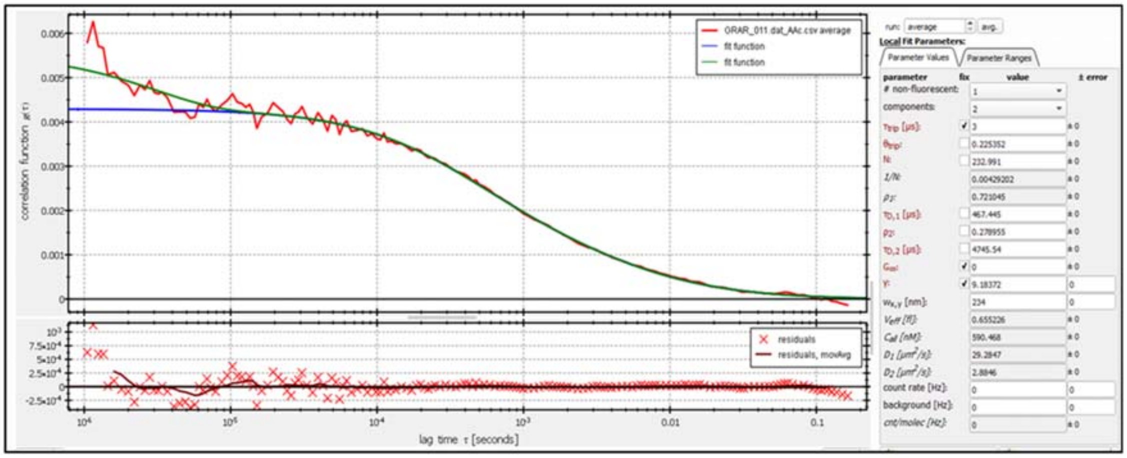


**f** EGFP-RARα + $H_2O_2$

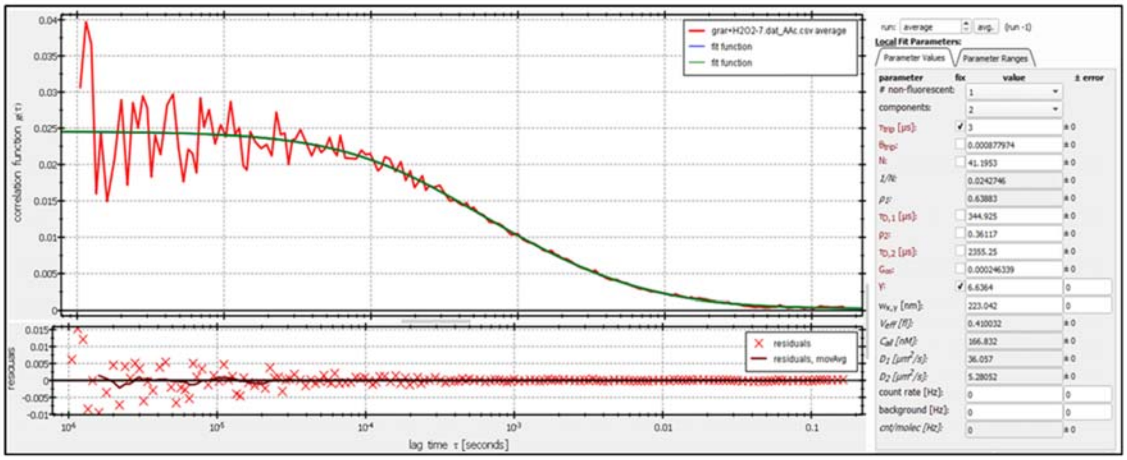


**Figure S10: Autocorrelation curves of EGFP dimer and EGFP-RARα in control and $H_2O_2$ / AM580 treated HeLa cells.** (**a-b**) Raw average autocorrelation curves from 10×8 s runs (red) fit to a model with one diffusion component, with (green) or w/o triplet term (blue) of the EGFP dimer used as control. Below, fit residuals, on the right, fit parameters are shown. (**c-f**) Autocorrelation curves of EGFP-RARα. Treatments with $H_2O_2$ (200 µM, 20 min) and/or AM580 RAR agonist (0.1 µM, 30 min) are indicated above the panels.

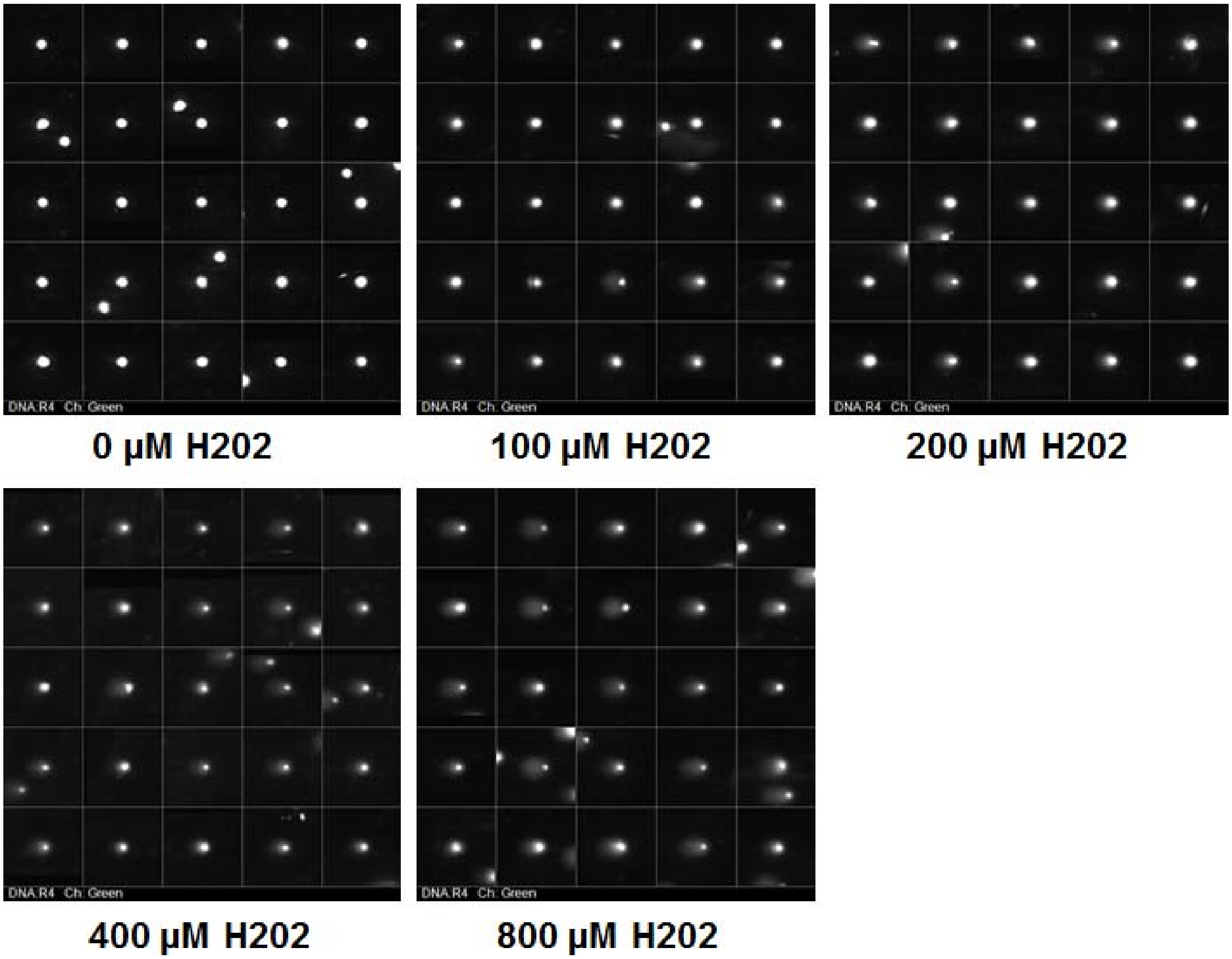


**Figure S11: Demonstration of nicking by $H_2O_2$ using alkaline comet assay.**

U2OS-2FP cells were treated with different concentrations of $H_2O_2$ (as indicated below the images) for 20 min at 37°C. DNA was stained with 5× Sybr Gold overnight at 4°C and samples imaged with a laser scanning cytometer.

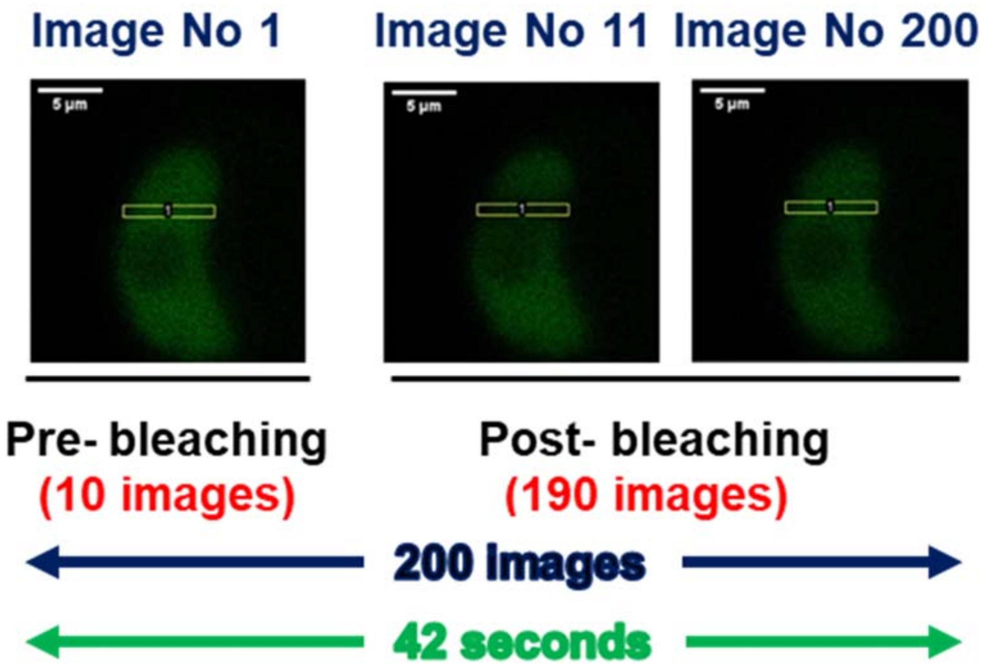


**Figure S12: Representative confocal microscopic images showing the applied FRAP settings**

Cells expressing EGFP-RARα before bleaching, directly after bleaching and at the end of the time series. Within 42 s, 200 images were collected as follows: 10 images before and 190 images after bleaching.

**Supplementary references**

1 Blair, R. H. *et al.* The HMGB1 C-Terminal Tail Regulates DNA Bending. *J Mol Biol* **428**, 4060-4072, doi:10.1016/j.jmb.2016.08.018 (2016).

2 Imre, L. *et al.* Nucleosome stability measured in situ by automated quantitative imaging. *Sci Rep* **7**, 12734, doi:10.1038/s41598-017-12608-9 (2017).

3 Nanasi, P., Jr. *et al.* Doxorubicin induces large-scale and differential H2A and H2B redistribution in live cells. *PLoS One* **15**, e0231223, doi:10.1371/journal.pone.0231223 (2020).
